# Explainable random forest–SHAP framework for Montreal phenotype–stratified prediction of one-year complications in Crohn’s disease

**DOI:** 10.3389/fphys.2026.1902465

**Published:** 2026-09-15

**Authors:** Kun Xia, Ying Shi, Xiaohan Huang, Yuwei Zhang, Liwei Suo, Shuntian Cai, Lingfeng Zhong, Dayi Zheng, Mengjie Lin, Sirui Wan, Linwei Wang, Jianlin Ren, Qingan Xia, Hongzhi Xu, Yanyun Fan

**Affiliations:** 1 Department of Gastroenterology, The National Key Clinical Specialty, Zhongshan Hospital of Xiamen University, School of Medicine, Xiamen University, Xiamen, Fujian, China; 2 Department of Health Technology and Informatics, The Hong Kong PolytechnicUniversity, Hong Kong, Hong Kong SAR, China; 3 Clinical Research Center for Gut Microbiota and Digestive Diseases of Fujian Province, Xiamen Key Laboratory of Intestinal Microbiome and Human Health, Xiamen, Fujian, China; 4 Department of Digestive Disease, Institute for Microbial Ecology, Xiamen University, Xiamen, Fujian, China; 5 Gastroenterology Department, First Affiliated Hospital of Jinan University, Guangzhou, Guangdong, China; 6 Department of Infectious Diseases, The Second Affiliated Hospital of Xiamen Medical College, Xiamen, Fujian, China; 7 Xiamen Gastroenterology Quality Control Center, Xiamen, Fujian, China; 8 Department of Pathology, Zhongshan Hospital of Xiamen University, School of Medicine, Xiamen University, Xiamen, Fujian, China; 9 Hospital Clínic–IDIBAPS, University of Barcelona, Barcelona, Spain; 10 Department of Pathology, TangShan GongRen Hospital, Tangshan, Hebei, China

**Keywords:** artificial intelligence, Crohn’ s disease, inflammatory bowel disease, machine learning, prognosis factors

## Abstract

**Background:**

Early identification of phenotype-stratified, short-term complication risk remains a critical unmet need in Crohn’s disease (CD). Existing models focus on single outcomes and lack interpretability, limiting clinical use. We developed a clinically interpretable machine-learning framework to enable Montreal phenotype–stratified prediction of three one-year complication categories: bowel resection (Task 1), perianal complications (Task 2), and abdominal complications (Task 3).

**Methods:**

Data from 370 patients across two clinical centers were pooled for model development and evaluated using stratified 10-fold cross-validation. Within each fold, data preprocessing, LASSO feature selection, SMOTE-Tomek resampling, model optimization, calibration, and threshold determination were restricted to the training data. Seven machine-learning algorithms were compared in terms of discrimination, calibration, with decision-curve analysis used to explore potential net benefit. SHapley Additive exPlanations (SHAP) were used for model interpretation, and nomograms, heatmaps, and a web-based calculator were generated as exploratory visualizations of model-derived risk estimates rather than as clinical decision-support tools. Phenotype-stratified and non-linear associations between CDAI and complication risk were further analyzed within the Montreal framework.

**Results:**

Within one year after discharge, bowel resection, perianal complications, and abdominal complications occurred in 70 (18.9%), 142 (38.4%), and 84 (22.7%) patients, respectively. Random Forest showed the most consistent overall performance, achieving an AUROC of 0.86 (95% CI, 0.82–0.90) for bowel resection, 0.71 (95% CI, 0.64–0.78) for perianal complications, and 0.73 (95% CI, 0.69–0.78) for abdominal complications. SHAP identified distinct predictors across outcomes: disease behavior, nutritional support therapy, and disease duration for bowel resection; prior perianal complications, extraintestinal manifestations, and CDAI for perianal complications; and prior abdominal complications, nutritional support therapy, and Montreal L3 phenotype for abdominal complications. Receipt of NST was interpreted as a clinical marker of nutritional depletion and greater disease burden rather than as a causal risk factor. Montreal-based stratification revealed marked heterogeneity, with isolated small-bowel disease associated with higher risks of bowel resection and abdominal complications, and isolated colonic disease with the highest burden of perianal complications.

**Conclusion:**

The interpretable RF–SHAP framework showed potential for Montreal phenotype–stratified prediction of one-year complication risk in CD and inform future evaluation of risk stratification and phenotype-informed management.

## Introduction

Crohn’s disease (CD) is a chronic, relapsing inflammatory bowel disease (IBD) driven by complex interactions among genetic, environmental, and microbiome–related factors, with a projected population prevalence approaching 1% (Ng et al., 2017; King et al., 2020; Dolinger et al., 2024). Characterized by profound clinical and biological heterogeneity, CD follows a highly variable disease course ranging from mild inflammatory activity to severe, progressive tissue destruction. This heterogeneity critically shapes both short–term and long–term prognosis, as cumulative bowel damage frequently leads to functional impairment, reduced quality of life, and a substantial healthcare burden due to recurrent hospitalizations and surgical interventions (Calvet et al., 2025). Furthermore, anatomical disease distribution represents a critical determinant of clinical phenotype, influencing symptom burden, disease severity, and susceptibility to specific complications (Baumgart and Sandborn, 2012; Lamb et al., 2019). While a proportion of patients experiences an indolent course, those with defined risk factors are prone to an aggressive disease trajectory characterized by rapid progression and irreversible disability. Consequently, the implementation of personalized management strategies, specifically the early identification of high–risk individuals and accurate risk stratification, is imperative to enable timely interventions and mitigate adverse outcomes.

To facilitate early identification of disease progression, increasing attention has been directed toward the application of artificial intelligence (AI) for risk stratification of one–year complications in patients with CD after discharge (Aygun et al., 2024; Xia et al., 2025). Accordingly, a growing body of research has leveraged large–scale electronic health records (EHR) or other multimodal data to develop machine learning (ML) models for predicting disease progression and complications risk (Wang et al., 2024; Lev-Tzion et al., 2025; Wang et al., 2025; Xiang et al., 2025; Xia et al., 2025). Some studies have also incorporated interpretable methods to improve the transparency of model predictions.

Despite these advances, independent external validation remains limited among existing models, restricting the assessment of their transportability across clinical settings. Furthermore, prior studies have largely focused on individual complications using limited demographic characteristics and routinely available laboratory variables (Nguyen et al., 2022; Wang et al., 2024; Wang et al., 2025; Xia et al., 2025). These differences in study populations, predictors, and outcome definitions may limit direct comparison and broader clinical application. Relatively few studies have evaluated bowel resection, perianal complications, and abdominal complications within a common prognostic framework and a consistent one-year time frame (Xiang et al., 2025). Therefore, the present study extends previous work by integrating these three outcomes with SHAP-based interpretation and exploratory Montreal phenotype–stratified analyses.

In this study, we used pooled EHR data from 370 patients across two clinical centers to develop an interpretable Random Forest (RF) framework for predicting one-year CD-related complications. Using the Least Absolute Shrinkage and Selection Operator (LASSO) for feature selection, the model was developed on a parsimonious set of routinely available clinical variables, targeting distinct one–year outcomes, including ileocecal resection and complex complications such as perianal or abdominal events. Beyond predictive performance, we further incorporated Montreal phenotype–based stratification to elucidate the relationship between anatomical disease patterns and prognosis. Collectively, these findings provide an interpretable framework for identifying patients at increased risk and support further investigation of phenotype-informed risk assessment in CD.

## Methods

### Study population

Our retrospective two-center cohort included 370 patients with CD from Zhongshan Hospital of Xiamen University (XMUZSH) (Sep 2013–Nov 2022) and the First Affiliated Hospital of Jinan University (JNFH) (Jan 2023–Nov 2023) with a minimum follow–up of one year. Data from the two centers were pooled before model development and cross-validation. **Figure 1**, **Table 1** and **Supplementary Tables 1**-**3** summarized the demographic and clinical profiles for four key comparisons: the inter–cohort analysis (XMUZSH vs. JNFH) and the intra–cohort stratifications based on the occurrence of bowel resection, perianal, and abdominal complications. This retrospective study was conducted in accordance with the Declaration of Helsinki. The requirement for informed consent was waived due to the retrospective design. The studies involving human participants were reviewed and approved by the local Ethical Review Board of Zhongshan Hospital of Xiamen University (XMZSYY–AF–SC–12–03).

**Figure 1:**
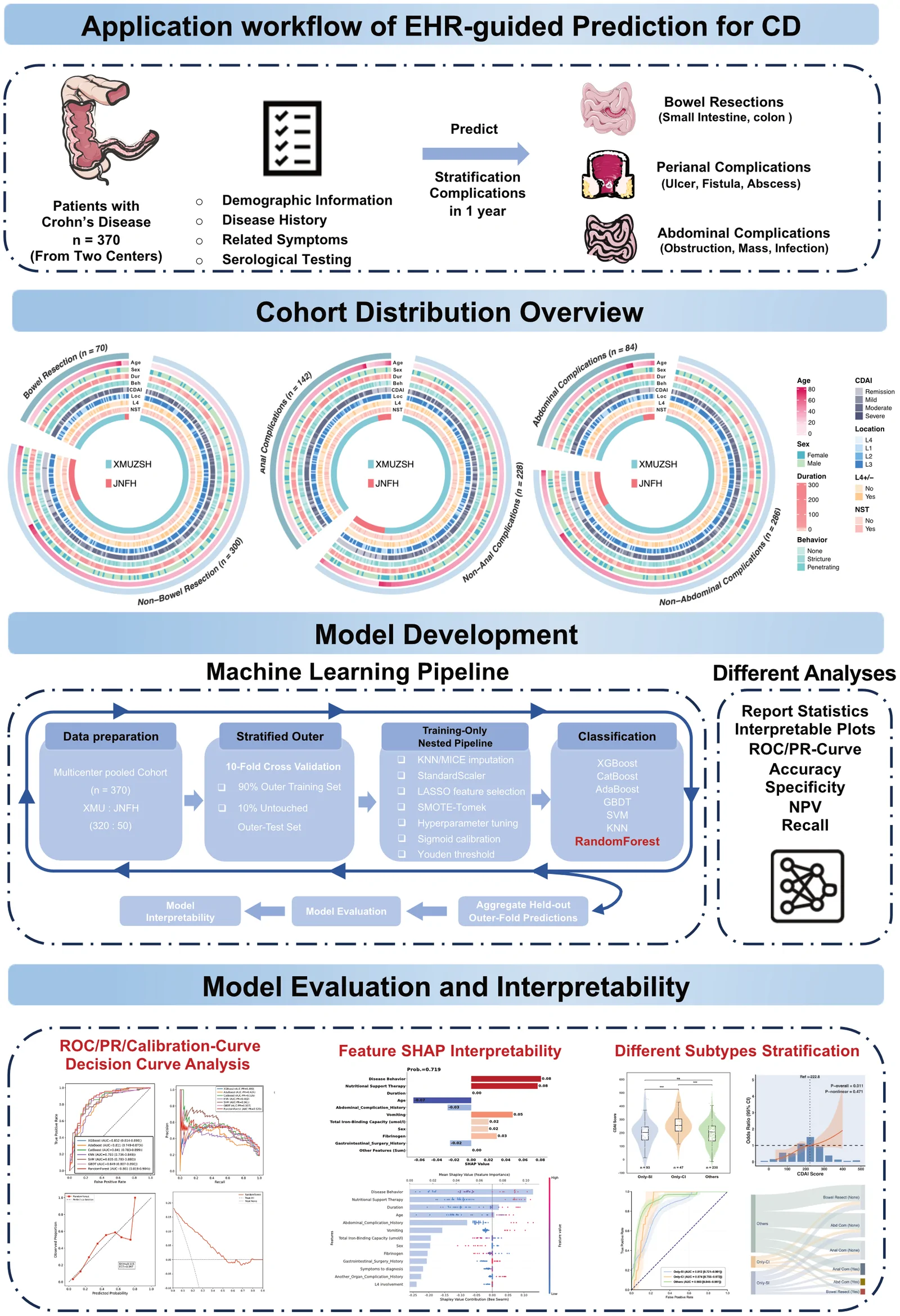
Study design concept. This study integrated fold-specific LASSO feature selection and SMOTE-Tomek resampling within a stratified 10-fold cross-validation framework to identify the optimal classifier among seven candidate algorithms. Model performance was evaluated using multidimensional metrics, followed by SHAP-based interpretation and the development of exploratory risk visualizations for the three one-year outcomes.

**Table 1:** Comparison of baseline demographic and clinical characteristics from different hospitals in one-year follow-up.

Variable	Total	Zhong Shan Hospital of Xiamen	Ji Nan University	χ2 /Z	P
	(N =370)	(N = 320)	(N = 50)		
Sex, n (%)					
Male	268 (72.4)	228 (71.3)	40 (80.0)	1.658^a^	0.198
Female	102 (27.6)	92 (28.7)	10 (20.0)		
Age, (Years)	28.0 (22.0, 35.2)	28.0 (22.0, 35.0)	29.5 (20.0, 36.5)	-0.060^b^	0.952
Course of diagnosis, (Months)	0.0 (0.0, 9.0)	0.0 (0.0, 12.0)	0.0 (0.0, 1.0)	-3.211^b^	0.001^*^
Symptoms to diagnosis, (Months)	6.0 (0.5, 24.0)	6.0 (0.0, 24.0)	6.0 (2.8, 17.0)	-1.262^b^	0.207
Duration, (Years)	12.0 (4.0, 45.0)	13.0 (5.0, 48.0)	8.5 (3.0, 17.0)	-2.913^b^	0.004^*^
Age of onset, (Years)	26.0 (20.0, 33.0)	26.0 (20.0, 33.0)	28.5 (18.0, 35.2)	-0.409^b^	0.683
BMI, (kg/m^2^)	19.0 (17.3, 21.5)	19.0 (17.3, 21.5)	18.6 (17.2, 21.6)	-0.252^b^	0.801
Smoking history, n (%)					
No	323 (87.3)	279 (87.2)	44 (88.0)	0.026^a^	0.873
Yes	47 (12.7)	41 (12.8)	6 (12.0)		
Alcohol consumption history, n (%)					
No	340 (91.9)	293 (91.6)	47 (94.0)	0.095^a^	0.758
Yes	30 (8.1)	27 (8.4)	3 (6.0)		
Hepatitis B, n (%)					
No	333 (90.0)	286 (89.4)	47 (94.0)	1.028^a^	0.311
Yes	37 (10.0)	34 (10.6)	3 (6.0)		
Abdominal_history, n (%)					
No	192 (51.9)	157 (49.1)	35 (70.0)	7.594^a^	0.006^*^
Yes	178 (48.1)	163 (50.9)	15 (30.0)		
Disease behavior, n (%)					
No-stricturing & No-penetrating	113 (30.5)	90 (28.1)	23 (46.0)	9.151^a^	0.010^*^
Stricturing	173 (46.8)	159 (49.7)	14 (28.0)		
penetrating	84 (22.7)	71 (22.2)	13 (26.0)		
Lesion location, n (%)					
L1	55 (14.9)	45 (14.1)	10 (20)	21.539^a^	<0.001^*^
L2	47 (12.7)	44 (13.8)	3 (6)		
L3	137 (37)	106 (33.1)	31 (62)		
L4	7 (1.9)	7 (2.2)	0 (0)		
Merge	124 (33.5)	118 (36.9)	6 (12)		
Diarrhea, n (%)					
No	103 (27.8)	91 (28.4)	12 (24.0)	0.424^a^	0.515
Yes	267 (72.2)	229 (71.6)	38 (76.0)		
Abdominal pain, n (%)					
No	57 (15.4)	53 (16.6)	4 (8.0)	2.433^a^	0.119
Yes	313 (84.6)	267 (83.4)	46 (92.0)		
Abdominal bloating, n (%)					
No	282 (76.2)	252 (78.8)	30 (60.0)	8.387^a^	0.004^*^
Yes	88 (23.8)	68 (21.3)	20 (40.0)		
Gastrointestinal bleeding, n (%)					
No	255 (68.9)	233 (72.8)	22 (44.0)	16.759^a^	<0.001^*^
Yes	115 (31.1)	87 (27.2)	28 (56.0)		
Weight loss, n (%)					
No	218 (58.9)	184 (57.5)	34 (68.0)	1.970^a^	0.160
Yes	152 (41.1)	136 (42.5)	16 (32.0)		
Vomiting, n (%)					
No	321 (86.8)	277 (86.6)	44 (88.0)	0.078^a^	0.780
Yes	49 (13.2)	43 (13.4)	6 (12.0)		
Nausea, n (%)					
No	312 (84.3)	270 (84.4)	42 (84.0)	0.005^a^	0.946
Yes	58 (15.7)	50 (15.6)	8 (16.0)		
Fever, n (%)					
No	302 (81.6)	261 (81.6)	41 (82.0)	0.006^a^	0.941
Yes	68 (18.4)	59 (18.4)	9 (18.0)		
Anemia, n (%)					
None	160 (43.2)	137 (42.8)	23 (46.0)	-0.244^b^	0.807
Mild	155 (41.9)	139 (43.4)	16 (32.0)		
Moderate	50 (13.5)	40 (12.5)	10 (20.0)		
Severe	5 (1.4)	4 (1.3)	1 (2.0)		
Poor appetite, n (%)					
No	273 (73.8)	237 (74.1)	36 (72.0)	0.095^a^	0.758
Yes	97 (26.2)	83 (25.9)	14 (28.0)		
Fatigue, n (%)					
No	303 (81.9)	259 (80.9)	44 (88.0)	1.455^a^	0.228
Yes	67 (18.1)	61 (19.1)	6 (12.0)		
Another organ complication history, n (%)					
No	327 (88.4)	281 (87.8)	46 (92.0)	0.738^a^	0.390
Yes	43 (11.6)	39 (12.2)	4 (8.0)		
Abdominal complication history, n (%)					
No	308 (83.2)	268 (83.8)	40 (80.0)	0.436^a^	0.509
Yes	62 (16.8)	52 (16.3)	10 (20.0)		
Anal complication history, n (%)					
No	162 (43.8)	123 (38.4)	39 (78.0)	27.499^a^	<0.001^*^
Yes	208 (43.8)	197 (61.6)	11 (22.0)		
Frequency of Loose Stools	10.0 (0.0, 21.0)	10.0 (0.0, 20.0)	14.0 (7.0, 21.0)	-2.727^b^	0.006^*^
Abdominal Pain Severity (0-3)	7.0 (2.0, 14.0)	10.0 (7.0, 14.0)	1.0 (1.0, 2.0)	-7.388^b^	<0.001^*^
General Condition (0-4)	14.0 (7.0, 17.0)	14.0 (11.0, 18.0)	2.0 (1.0, 2.0)	-11.415^b^	<0.001^*^
Extramural complications, n (%)					
0	155 (41.9)	111 (34.7)	44 (88.0)	-6.714^a^	<0.001^*^
1	130 (35.1)	125 (39.1)	5 (10.0)		
2	72 (19.5)	71 (22.2)	1 (2.0)		
3	11 (3.0)	11 (3.4)	0 (0.0)		
4	2 (0.5)	2 (0.6)	0 (0.0)		
Opioid antidiarrheal medications, n (%)					
No	369 (99.7)	320 (100.0)	49 (98.0)	1.142^a^	0.135
Yes	1 (0.3)	0 (0.0)	1 (2.0)		
Reduced Hematocrit	2.0 (0.0, 5.9)	2.1 (0.0, 5.7)	1.9 (-2.6, 7.5)	-0.605^b^	0.546
(Standard - Actual) / Standard Weight	13.0 (2.9, 22.2)	12.7 (3.1, 22.3)	14.1 (1.6, 22.0)	-0.131^b^	0.896
CDAI Score	222.8 (153.3, 260.2)	229.8 (179.3, 267.2)	79.0 (51.0, 110.5)	-9.602^b^	<0.001^*^
Activity, n (%)					
Remission	87 (23.5)	43 (13.4)	44 (88.0)	133.677^a^	<0.001^*^
Active	283 (76.5)	277 (86.6)	6 (12.0)		
WBC (×10^9)	6.6 (5.2, 8.7)	6.5 (5.1, 8.5)	8.1 (5.7, 10.4)	-2.650^b^	0.008^*^
RBC (×10^12)	4.5 (4.0, 5.0)	4.5 (4.0, 5.0)	4.7 (4.2, 5.0)	-1.733^b^	0.083
HB (g/L)	117.5 (102.0, 133)	117.5 (102.0, 132.0)	118.5 (98.5, 135.0)	-0.065^b^	0.948
MCV (fL)	83.9 (78.0, 88.0)	84.2 (78.9, 88.0)	81.7 (72.9, 88.1)	-1.801^b^	0.072
MCH (Pg)	26.9 (24.2, 28.5)	27.0 (24.3, 28.6)	25.9 (22.6, 28.4)	-1.957^b^	0.050^*^
MCHC (g/L)	318.4 (307.0, 329.0)	319.0 (307.0, 329.0)	315.3 (308.5, 324.8)	-0.725^b^	0.469
HCT (%)	36.9 (32.8, 40.9)	36.9 (33.2, 40.7)	37.5 (31.7, 42.6)	-0.218^b^	0.827
PLT (×10^9)	325.5 (256.8, 404.0)	325.5 (258.0, 403.8)	328.9 (250.3, 409.9)	-0.143^b^	0.886
ESR (mm/h)	26.6 (15.8, 47.7)	26.3 (15.0, 46.5)	40.0 (21.8, 56.2)	-2.181^b^	0.029^*^
CRP (mg/L)	17.1 (5.0, 51.1)	17.0 (5.0, 45.7)	26.2 (5.6, 65.2)	-1.349^b^	0.177
TP (g/L)	69.7 (63.2, 75.1)	70.2 (63.9, 75.2)	68.2 (61.4, 72.6)	-2.042^b^	0.041^*^
ALB (g/L)	37.0 (32.9, 40.6)	37.0 (32.9, 40.6)	37.0 (32.4, 40.7)	-0.092^b^	0.926
Urea Nitrogen (mmol/L)	3.7 (3.0, 4.6)	3.7 (3.0, 4.6)	3.8 (3.0, 5.0)	-0.863^b^	0.388
Serum Potassium (mmol/L)	3.9 (3.6, 4.2)	3.9 (3.6, 4.1)	4.1 (3.7, 4.3)	-2.918^b^	0.004^*^
Uric Acid (μmol/L)	333.8 (266.5, 393.5)	330.1 (262.0, 390.0)	354.6 (307.5, 411.6)	-1.879^b^	0.060
HDL (mmol/L)	1.0 (0.8, 1.1)	1.0 (0.8, 1.1)	1.0 (0.7, 1.3)	-0.467^b^	0.640
Cholesterol (mmol/L)	3.5 (3.0, 4.1)	3.5 (3.0, 4.1)	3.9 (3.2, 4.5)	-2.624^b^	0.009^*^
Triglycerides (mmol/L)	0.9 (0.7, 1.2)	0.9 (0.7, 1.2)	0.9 (0.8, 1.1)	-0.702^b^	0.482
Folic Acid (nmol/L)	11.5 (7.6, 16.2)	12.1 (7.9, 16.7)	9.5 (7.3, 11.9)	-2.879^b^	0.004^*^
VitB12 (pmmol/L)	310.7 (260.7, 384.8)	299.0 (251.2, 379.2)	375.3 (327.5, 412.3)	-4.462^b^	<0.001^*^
Fe (umol/L)	8.4 (5.2, 11.8)	8.8 (5.1, 11.8)	7.5 (5.4, 11.4)	-0.400^b^	0.689
Total Iron-Binding Capacity (umol/L)	48.9 (42.0,54.4)	48.0 (41.2,54.1)	52.9 (47.2,57.2)	-3.512^b^	<0.001^*^
Serum Ferritin (ng/mL)	128.0 (66.5, 205.1)	122.4 (63.0, 202.8)	156.6 (119.5, 219.5)	-2.446^b^	0.014^*^
Serum Transferrin (g/L)	2.0 (1.8, 2.2)	2.0 (1.8, 2.2)	2.0 (1.7, 2.1)	-1.089^b^	0.276
Soluble Transferrin Receptor (mg/L)	1.3 (1.1, 1.6)	1.2 (1.1, 1.5)	31.7 (1.8, 31.8)	-8.734^b^	<0.001^*^
Fecal occult blood, n (%)					
No	74 (20.0)	57 (17.8)	17 (34.0)	7.082^a^	0.008^*^
Yes	296 (80.0)	263 (82.2)	33 (66.0)		
Calprotectin, n (%)					
No	11 (3.0)	11 (3.4)	0 (0.0)	0.780^a^	0.377
Yes	359 (97.0)	309 (96.6)	50 (100.0)		
Fibrinogen (mg/L)	4.0 (3.2, 4.7)	3.9 (3.2, 4.7)	4.3 (3.3, 5.3)	-1.644^b^	0.100
D-Dimer(mg/L)	0.3 (0.2, 0.6)	0.3 (0.2, 0.6)	0.4 (0.2, 0.8)	-2.486^b^	0.013^*^
Total enteral nutrition, n (%)					
No	268 (72.4)	230 (71.9)	38 (76.0)	0.368^a^	0.544
Yes	102 (27.6)	90 (28.1)	12 (24.0)		

Continuous data exhibiting a non-normal distribution are summarized as medians (IQR), while categorical variables are presented as frequencies and percentages.

*p < 0.05. ^a^: Calculated via Chi-square test. ^b^: Calculated via Mann-Whitney U test.

Patient inclusion criteria were as follows: (1) age 18–70 years; (2) a confirmed diagnosis of CD according to the 2021 ECCO guideline and the 2023 Chinese national clinical practice guideline (Gordon et al., 2023; Inflammatory Bowel Disease Group et al., 2024); (3) a minimum follow–up duration of 12 months; (4) availability of the demographic characterize and clinical data required for model development.

### Data collection and processing

The prediction time origin was discharge from the index hospitalization, and only information documented by that time was eligible for model development. Comprehensive data collection encompassed a comprehensive profile of demographics data and clinical characteristics, including disease history (smoking history, alcohol and Hepatitis B), phenotype (behavior and lesion location) and symptomatology (fever, fatigue, diarrhea, gastrointestinal bleed, vomitus, etc.), alongside a broad panel of laboratory biomarkers reflecting inflammatory and nutritional support therapy (NST). Receipt of NST during the index hospitalization was recorded as a treatment-related predictor available by discharge. The laboratory indicators mainly included Red Blood Cell Count (RBC), Hemoglobin (HB), Mean Corpuscular Volume (MCV), C–reactive Protein (CRP), Uric Acid, Folic Acid, VitB12, Fibrinogen, Total Iron–Binding Capacity and other routinely available measurements.

Disease location was stratified according to the Montreal classification (Satsangi, 2006), re–coded into six mutually exclusive categories to capture specific topographic patterns. Standard phenotypes included ileal (L1), colonic (L2), ileocolonic (L3), and isolated upper gastrointestinal (L4) disease. We classified disease behavior into three exclusive categories: non–stricturing and non–penetrating (B1); stricturing (B2); and penetrating (B3). This stratification strategy facilitates consistent phenotypic reporting and has been rigorously substantiated by the literature (Satsangi, 2006). Univariable logistic regression analyses were first performed to screen potential risk factors for each specific complication outcome. Variables demonstrating statistical significance (P < 0.05) were subsequently entered into a multivariable logistic regression model to identify independent predictors (**Figure 2**).

**Figure 2:**
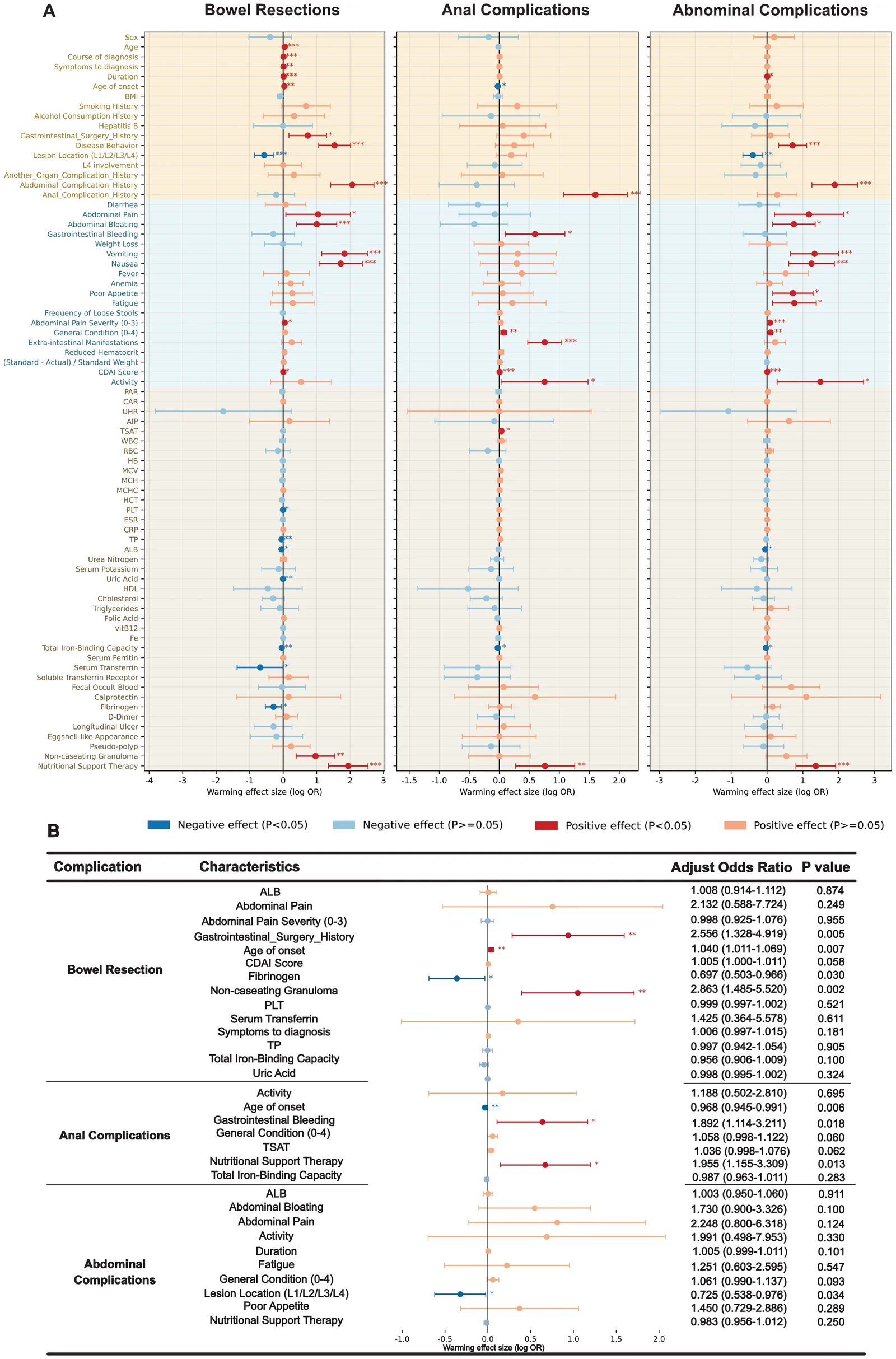
Identification of risk factors and independent predictors for specific complication subtypes. aOR, adjust odds ratio. CI, confidence interval. VIF, variance inflation factor. **(A)** Univariable logistic regression analysis screening candidate variables associated with bowel resection, perianal lesions, and abdominal complications. Red and blue markers indicate positive and inverse associations, respectively (p<0.05). **(B)** Multivariable logistic regression analysis identifying independent predictors. Candidate variables (VIF ≤ 10) were adjusted for confounders. The forest plots display aOR with 95% CI.

We established a leakage-safe preprocessing pipeline within the nested cross-validation framework (**Figure 1**). Variables exhibiting a missing rate ≥ 20% were excluded. Within each training partition, categorical and continuous missing values were handled using fold-fitted K–nearest Neighbors (KNN) and Multiple Imputation by Chained Equations (MICE) imputation, respectively (**Supplementary Table 4**). The fitted imputers were subsequently applied without refitting to the corresponding validation or outer-test data. Standardization parameters were estimated from training data only. LASSO feature selection was repeated within each training fold, allowing the selected features to vary across folds. To address class imbalance, SMOTE-Tomek resampling was performed exclusively on training partitions and was never applied to validation or outer-test observations (Lemaıtre and Nogueira, 2017).

### Training labels

The three one-year outcomes were defined as follows: (1) Bowel Resection (comprising small bowel, colorectal, or multi–segment resections); (2) Perianal Complications (including hematochezia explicitly linked to perianal pathology, fissures, ulcers, fistulas, and perianal abscesses); and (3) Abdominal Complications (defined by the presence of abdominal masses, obstruction, or intestinal infection). Presence of any constituent event within the one–year follow–up qualified as a positive outcome.

### Model construction and training

We conducted a comprehensive benchmarking analysis using seven distinct machine learning classifiers—RF, XGBoost, AdaBoost, CatBoost, Gradient–based Decision Tree (GBDT), Support Vector Machine (SVM), and KNN—to predict the one–year risk of the aforementioned three types of outcomes. To evaluate model performance and reduce the risk of overfitting, we used stratified 10-fold cross-validation. In each outer fold, 90% of the pooled cohort was used for model development and the remaining 10% was reserved for performance evaluation. All model-development procedures were restricted to the training data. Hyperparameters were selected through training-only cross-validation, using RandomizedSearchCV for candidate algorithms. Probability calibration was subsequently performed using the sigmoid method within the training data. Finally, a fold-specific diagnostic threshold was determined by maximizing Youden’s J statistic based on out-of-fold predictions from the training set. The resulting model and threshold were then applied unchanged to the corresponding held-out test fold. For the specific model parameters used in this study, please refer to **Supplementary Methods S1**.

### Model performance evaluation and interpretability analyses

The predictive performance of the ML models was comprehensively assessed through a multi–metric approach. Model performance was evaluated in terms of discrimination, calibration, and potential net benefit within the internally cross-validated cohort. Discrimination was assessed using the area under the receiver operating characteristic curve (AUROC) and the area under the precision–recall curve (AUPRC). Calibration was evaluated using calibration curves and the Brier score, while decision-curve analysis (DCA) was used to estimate net benefit across a range of threshold probabilities (**Supplementary Figure 3**). Threshold-dependent metrics included accuracy, recall, specificity, negative predictive value (NPV), and F1 score (**Figure 3**, **Table 2**). All performance estimates were derived from held-out outer-fold predictions. Discrimination and calibration were assessed using the predicted probabilities from the untouched outer folds, whereas threshold-dependent metrics were calculated using the corresponding thresholds derived from training-set out-of-fold predictions.

**Figure 3:**
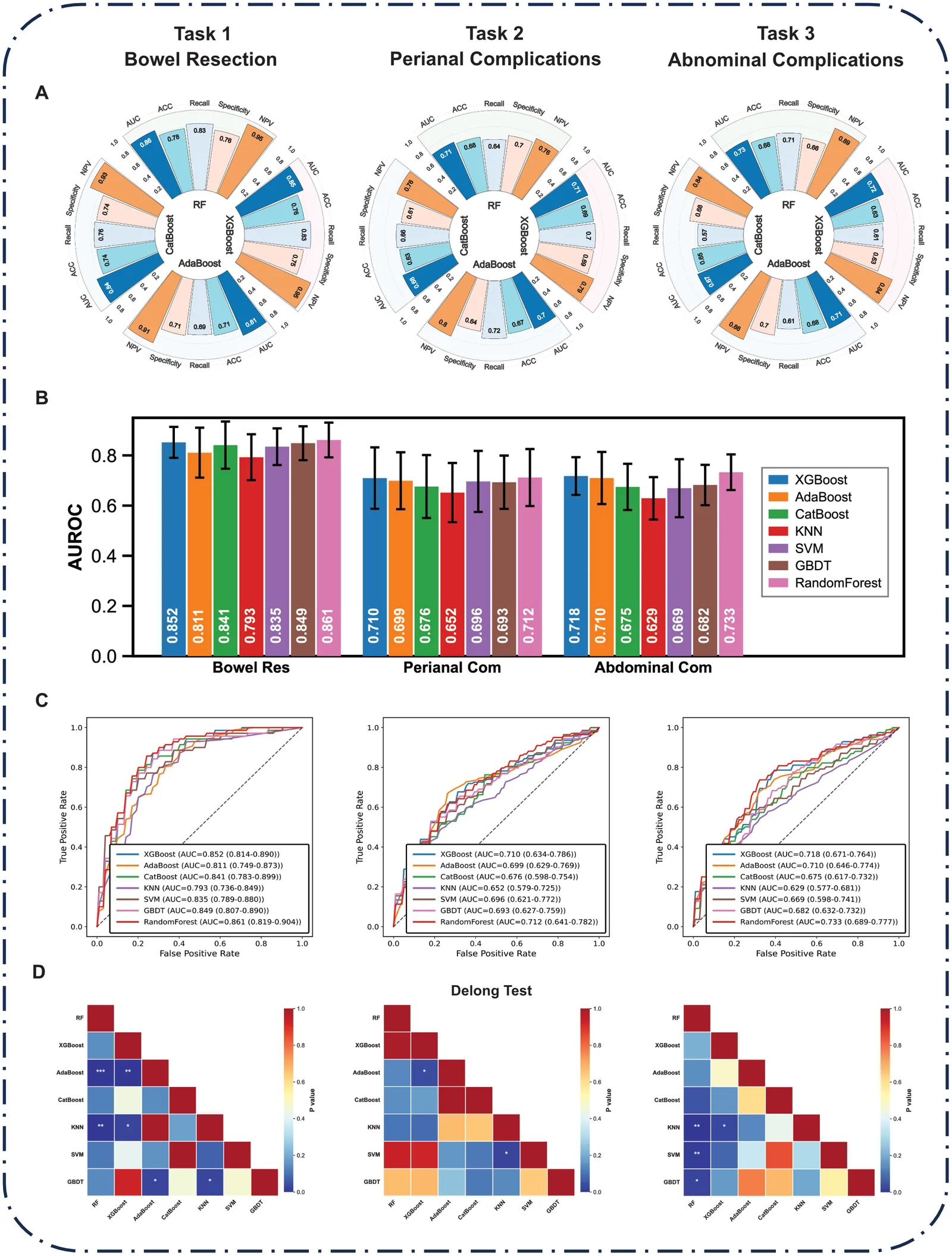
Comparison of seven ML models for predicting three types of complications using held-out outer-fold predictions. **(A)** Radar plots of performance metrics for Task 1 (Bowel Resection), Task 2 (Perianal Complications), and Task 3 (Abdominal Complications). **(B)** Mean AUROC with 95% CI for each model across the three tasks. **(C)** ROC curves for the seven models in Tasks 1–3. **(D)** Pairwise AUROC comparisons using the DeLong test (lower-triangular heatmaps; P<0.05, *P<0.01, **P<0.001). AUROC, area under the receiver operating characteristic curve.

**Table 2:** Internally cross-validated performance of the machine-learning models in the pooled two-center cohort.

Task	Different methods	AUROC (95% CI)	AUPRC(95% CI)	Brier Score (95% CI)	Accuracy (95% CI)	Recall(95%CI)	Specificity (95%CI)	NPV (95% CI)
Bowel Resection	RF	0.86 (0.82-0.90)	0.58 (0.51-0.66)	0.115 (0.095-0.135)	0.78 (0.73-0.82)	0.83 (0.75-0.91)	0.76 (0.71-0.82)	0.95 (0.93-0.97)
	XGBoost	0.85 (0.81-0.89)	0.56 (0.48-0.65)	0.120 (0.098-0.142)	0.76 (0.71-0.81)	0.83 (0.72-0.94)	0.75 (0.68-0.82)	0.95 (0.93-0.98)
	CatBoost	0.84 (0.78-0.90)	0.58 (0.50-0.66)	0.117 (0.097-0.139)	0.74 (0.70-0.79)	0.76 (0.65-0.87)	0.74 (0.68-0.79)	0.93 (0.90-0.96)
	AdaBoost	0.81 (0.75-0.87)	0.51 (0.42-0.61)	0.131 (0.111-0.153)	0.71 (0.64-0.77)	0.69 (0.51-0.86)	0.71 (0.62-0.80)	0.91 (0.87-0.96)
	KNN	0.79 (0.74-0.85)	0.47 (0.39-0.56)	0.128 (0.108-0.151)	0.73 (0.68-0.77)	0.69 (0.51-0.86)	0.74 (0.66-0.81)	0.92 (0.88-0.96)
	SVM	0.83 (0.79-0.88)	0.62 (0.57-0.68)	0.114 (0.094-0.136)	0.75 (0.71-0.80)	0.77 (0.66-0.88)	0.75 (0.70-0.79)	0.93 (0.90-0.97)
	GBDT	0.85 (0.81-0.89)	0.60 (0.52-0.68)	0.117 (0.097-0.139)	0.76 (0.72-0.81)	0.77 (0.64-0.90)	0.76 (0.72-0.81)	0.94 (0.90-0.97)
Perianal Complication	RF	0.71 (0.64-0.78)	0.61 (0.53-0.69)	0.212 (0.195-0.229)	0.68 (0.61-0.74)	0.64 (0.54-0.75)	0.70 (0.65-0.75)	0.76 (0.70-0.83)
	XGBoost	0.71 (0.63-0.79)	0.63 (0.54-0.71)	0.211 (0.191-0.233)	0.69 (0.64-0.75)	0.70 (0.60-0.80)	0.69 (0.63-0.76)	0.79 (0.74-0.85)
	CatBoost	0.68 (0.60-0.75)	0.60 (0.52-0.68)	0.219 (0.205-0.235)	0.63 (0.58-0.69)	0.66 (0.55-0.78)	0.61 (0.54-0.69)	0.76 (0.68-0.84)
	AdaBoost	0.70 (0.63-0.77)	0.59 (0.52-0.65)	0.217 (0.200-0.235)	0.67 (0.63-0.72)	0.72 (0.62-0.82)	0.64 (0.60-0.69)	0.80 (0.73-0.86)
	KNN	0.65 (0.58-0.72)	0.57 (0.50-0.64)	0.221 (0.206-0.238)	0.59 (0.55-0.64)	0.53 (0.39-0.67)	0.64 (0.56-0.72)	0.70 (0.63-0.76)
	SVM	0.70 (0.62-0.77)	0.62 (0.54-0.70)	0.210 (0.193-0.227)	0.65 (0.60-0.69)	0.60 (0.47-0.73)	0.68 (0.63-0.73)	0.75 (0.68-0.81)
	GBDT	0.69 (0.63-0.76)	0.62 (0.55-0.70)	0.215 (0.201-0.228)	0.66 (0.61-0.70)	0.64 (0.54-0.74)	0.67 (0.62-0.71)	0.76 (0.70-0.81)
Abdominal Complication	RF	0.73 (0.69-0.78)	0.52 (0.44-0.59)	0.160 (0.137-0.182)	0.68 (0.63-0.73)	0.71 (0.65-0.78)	0.66 (0.59-0.74)	0.89 (0.87-0.91)
	XGBoost	0.72 (0.67-0.76)	0.51 (0.44-0.59)	0.163 (0.140-0.187)	0.63 (0.55-0.70)	0.61 (0.52-0.70)	0.63 (0.53-0.74)	0.84 (0.81-0.88)
	CatBoost	0.67 (0.62-0.73)	0.46 (0.38-0.54)	0.166 (0.144-0.188)	0.65 (0.59-0.72)	0.57 (0.48-0.67)	0.68 (0.60-0.75)	0.84 (0.81-0.88)
	AdaBoost	0.71 (0.65-0.77)	0.47 (0.38-0.55)	0.165 (0.143-0.191)	0.68 (0.61-0.75)	0.61 (0.47-0.74)	0.70 (0.60-0.79)	0.86 (0.82-0.90)
	KNN	0.63 (0.58-0.68)	0.41 (0.34-0.49)	0.168 (0.146-0.191)	0.62 (0.58-0.67)	0.58 (0.50-0.67)	0.63 (0.58-0.69)	0.84 (0.81-0.87)
	SVM	0.67 (0.60-0.74)	0.43 (0.34-0.52)	0.168 (0.146-0.192)	0.61 (0.54-0.67)	0.58 (0.50-0.65)	0.62 (0.53-0.70)	0.83 (0.79-0.86)
	GBDT	0.68 (0.63-0.73)	0.46 (0.39-0.54)	0.166 (0.145-0.187)	0.63 (0.55-0.71)	0.61 (0.51-0.72)	0.64 (0.53-0.74)	0.84 (0.80-0.89)

Values are presented as the mean across the 10 held-out outer folds, with descriptive fold-based 95% intervals reflecting variation across the outer-fold partitions.

To enhance the transparency and interpretability of the best–performing model, we applied SHapley Additive exPlanations (SHAP). SHAP quantified the contribution of each predictor to the model output, providing global importance rankings and individual-level explanations. The plots illustrated the direction and magnitude of each feature’s contribution to model predictions. SHAP values were interpreted as model-based associations and not as evidence of biological causation. (**Figure 4**; **Supplementary Figure 5**). All interpretability analyses were executed using the SHAP Python library (v0.48.0).

**Figure 4:**
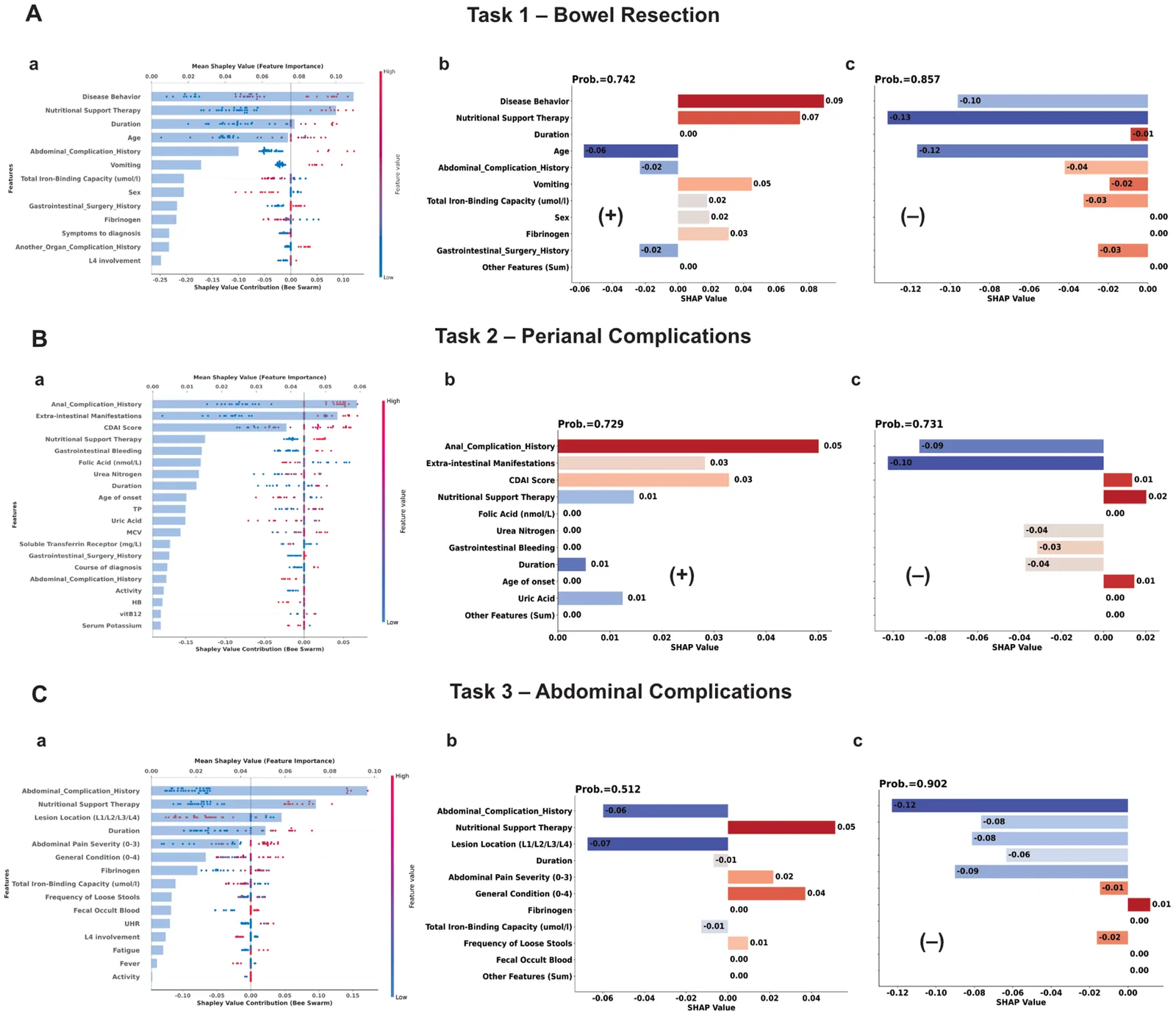
Interpretable SHAP plots for the RF models’ prediction results of three complications. The three subplots on the left are “Summary Plots and Feature Importance Ranking” across different complications in one–year follow–up period after discharge. SHAP values illustrate the directional impact and magnitude of clinical features on model output. Each discrete point represents an individual patient, with horizontal displacement quantifying the effect on predictive probability. These SHAP values describe model associations and do not indicate causal effects. Point color–coding denotes feature magnitude (red, high; blue, low), while vertical clustering reflects the distribution density of the cohort for each specific variable.

Finally, the most influential variables identified by SHAP for each outcome were selected to generate heatmaps and nomograms as exploratory visualizations of model-derived risk. These displays required further validation and were not intended to guide clinical treatment decisions (**Figure 5**; **Supplementary Figures 1**, **2**).

**Figure 5:**
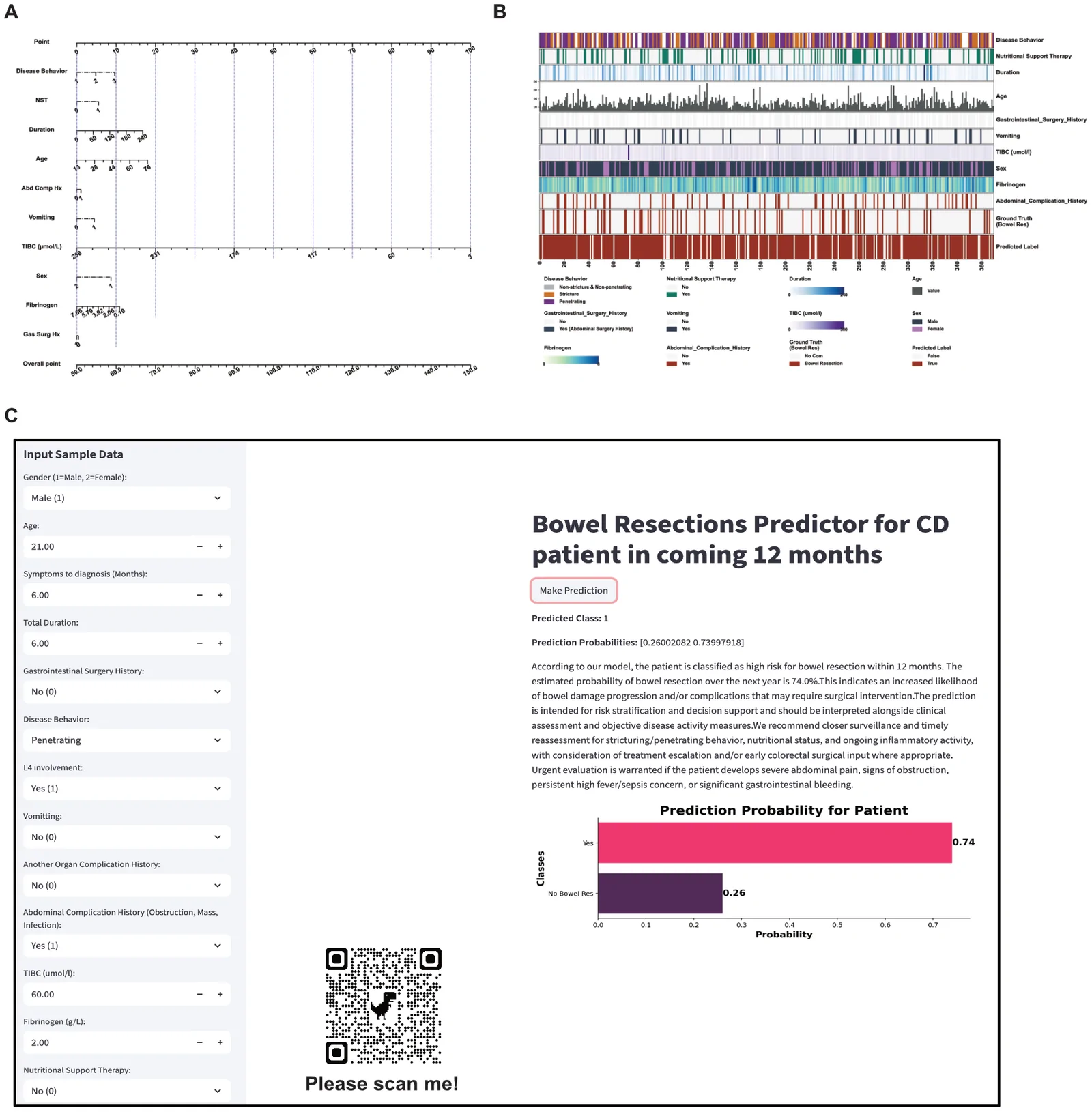
Exploratory visualization of model-derived risk estimates for bowel resection (Task 1). The figure presents an exploratory nomogram **(A)**, a heatmap based on the most influential variables identified by SHAP **(B)**, and a web-based calculator prototype **(C)**. A quantified score is assigned to each prognostic factor on the points scale. The total points, calculated by summing the scores from all factors, are subsequently translated into an individual estimate of positive risk. This tool enables the identification of patients with higher model-predicted risk who may warrant closer clinical assessment and follow-up. NST, nutritional support therapy.

### Location phenotypes and specific complication risks

For the exploratory analyses presented in **Figure 6**, patients were assigned to study-defined anatomical location groups derived from Montreal location information. These groups were not equivalent to the conventional L1/L2/L3/L4 categories used in the logistic regression analyses. For the initial three–group analysis, anatomical distribution was operationally grouped as “Only–SI” (isolated small–bowel involvement, defined as L1 alone or L1 with L4 modifier), “Only–CI” (isolated colonic disease involvement, L2 without L4), and “Others” (all remaining non–isolated or mixed Montreal location combinations). This grouping, used for visual analysis and model development, was distinct from formal Montreal subtypes. In the subsequent binary analysis, “Only–CI” and “Others” were merged into a single “Others” cohort, representing non–isolated small–bowel or mixed involvement. This stratification was employed to elucidate the influence of anatomical patterns on long–term prognosis, particularly cumulative bowel damage and phenotype–specific disease progression (**Figure 6**).

**Figure 6:**
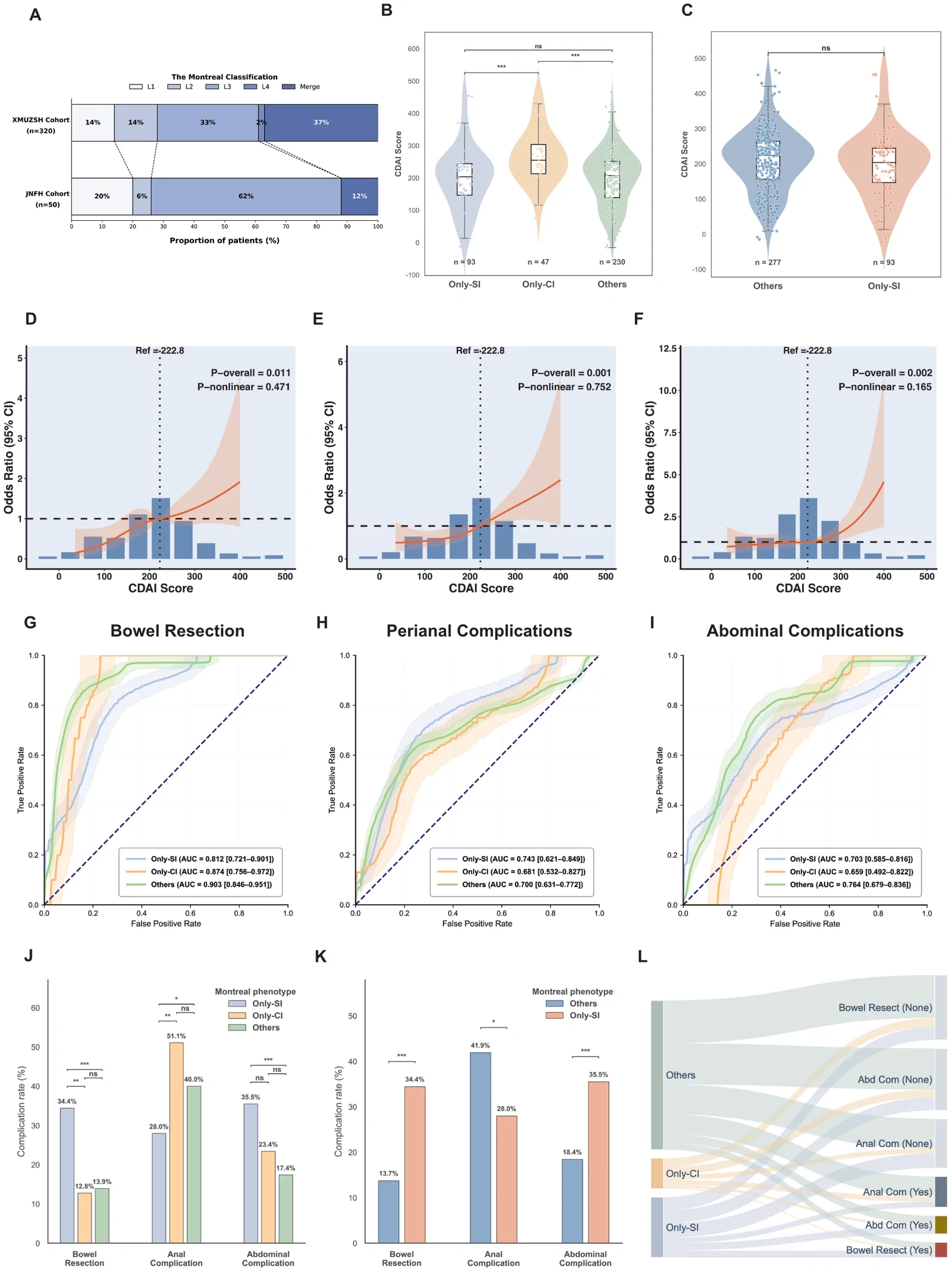
Association of study-defined anatomical phenotype groups derived from Montreal location information with disease activity and complication outcomes. Only–SI, Only Small Intestine. Only–CI, Only Colonic Intestine. **(A)** Stacked bar plots for illustrating the proportions of different Montreal phenotypes across the two cohorts. **(B, C)** Raincloud plots for phenotype distribution and comparative analysis of CDAI scores. Raincloud plots integrating probability density, boxplots, and individualized raw data points illustrate the symptomatic burden across different phenotypes, with statistical significance determined by Mann–Whitney tests. (ns, non–significant. *p < 0.05; **p < 0.01; ***p < 0.001). **(D–F)** Restricted cubic spline (RCS) analyses characterizing potential non–linear relationships between CDAI score and the risk of three complications. Curves show adjusted odds ratios with 95% CI, and histograms indicate CDAI distribution. The dashed line marks OR = 1.0 and the dotted line marks the reference CDAI (Ref). **(G–I)** ROC curves of the RF model across different phenotypes. **(J, K)** Clustered bar charts for one–year clinical complication rates stratified by phenotypic risk signatures. Clustered bar charts represent the proportions of patients experiencing specific complications, with statistical significance determined by Chi–square tests (ns, non–significant. *p < 0.05; **p < 0.01; ***p < 0.001). **(L)** Sankey diagram showing the association between baseline phenotypes and the occurrence of bowel resection, perianal and abdominal complications. The width of the bands represents the relative proportion of patients in each category.

### Statistics analysis

All analyses were performed utilizing Python (version 3.9.16). Categorical variables were presented as counts with percentages, and continuous data were reported as mean ± standard deviation or median with interquartile range, depending on the normality of the distribution. Differences between patients with and without complications were evaluated using the Mann–Whitney U test for non–normally distributed continuous variables and the chi–square test for categorical variables. To mitigate multicollinearity, predictors with a variance inflation factor (VIF) > 10 were excluded from multivariate logistic regression (MLR) models. Following stratification by Montreal classification and complication status, differences in CD Activity Index (CDAI) scores and complication proportions across subgroups were evaluated using Mann–Whitney U and Chi–square tests, respectively. Delong test was applied for pairwise comparison of AUROCs between models (**Figure 3D**). Furthermore, Spearman’s rank correlation analysis was performed to explore the relationship between CDAI scores and continuous laboratory biomarkers (**Supplementary Figure 4**). All statistical tests were two–sided, with significance defined as P < 0.05.

Benjamini–Hochberg correction was applied separately to the univariable analyses for each outcome and to the CDAI and complication-rate comparisons in **Figure 6**. The conventional Montreal classification used in **Figure 2** and the study-defined anatomical grouping used in **Figure 6** were analyzed separately.

## Results

### Baseline characteristics

A total of 370 patients with CD were enrolled from two tertiary medical centers (XMUZSH, n=320; JNFH, n=50). During the one–year follow–up post–discharge, 70 (18.9%) patients underwent small bowel or colonic resection. For disease–related complications, 142(38.4%) patients developed perianal complications (including ulcers, fistulas, and abscesses), while 84 (22.7%) presented with abdominal complications such as abdominal masses or intestinal infections (**Table 1**; **Supplementary Tables 1**-**3**).

### Identification of variables associated with specific complication subtypes

To identify specific risk factors for distinct complication phenotypes, we adopted a two–stage analysis strategy. First, univariate analysis was performed for screening, identifying candidate variables significantly associated with bowel resection (task 1), perianal lesions (task 2), and abdominal complications (P < 0.05) (**Figure 2A**). Subsequently, these candidate variables with VIF ≤10 were incorporated into a MLR model to adjust for confounding factors, thereby determining independent predictors.

In task 1, multivariable analysis revealed that a history of gastrointestinal surgery (adjusted odds ratio [aOR] 2.556, 95% CI: 1.328–4.919; P = 0.005) and the presence of non–caseating granuloma (aOR 2.863, 95% CI: 1.485–5.520; P = 0.002) were robust independent risk factors. Conversely, elevated fibrinogen levels exhibited an inverse association with the resection risk (aOR 0.697, 95% CI: 0.503–0.966; P = 0.030), suggesting a potential protective or baseline interaction effect. For task 2, NST (aOR 1.955, 95% CI: 1.155–3.309; P = 0.013) and a history of gastrointestinal bleeding (aOR 1.892, 95% CI: 1.114–3.211; P = 0.018) were independent risk factors. Notably, earlier age of onset was significantly associated with perianal involvement (aOR 0.968, 95% CI: 0.945–0.991; P = 0.006). Regarding task 3, while abdominal pain and bloating showed univariate associations, these were attenuated after multivariate adjustment.

When conventional Montreal disease location was treated as a categorical variable with L1 as the reference category, its overall association with abdominal complications was not statistically significant in the multivariable model (likelihood-ratio P = 0.108). In the category-specific comparisons, L3 was associated with lower odds than L1 (aOR 0.461, 95% CI 0.241–0.880; nominal P = 0.019), whereas L2 and L4 were not significantly different from L1. Given the nonsignificant overall test and the small number of patients classified as L4, these estimates should be interpreted cautiously.

### Evaluation of ML models in predicting complications

LASSO feature selection was repeated within each training fold of the nested cross-validation procedure. Consequently, the retained predictor set varied across outer folds rather than being fixed before cross-validation. Identifying these risk factors early is critical to mitigating the progressive bowel damage and disability characteristic of CD. As illustrated in **Figure 3** and **Table 2**, RF showed the most consistent overall performance across the three prediction tasks. It achieved the highest AUROC for bowel resection and abdominal complications and a joint-highest AUROC with XGBoost for perianal complications.

RF showed the strongest discrimination for bowel resection, with an AUROC of 0.86 (95% CI, 0.82–0.90), a recall of 0.83 (95% CI, 0.75–0.91), and an NPV of 0.95 (95% CI, 0.93–0.97). Its performance was more moderate for perianal (task 2) and abdominal complications (task 3), with AUROCs of 0.71 (95% CI, 0.64–0.78) and 0.73 (95% CI, 0.69–0.78), respectively. Full performance estimates are presented in **Table 2**.

As a sensitivity analysis, the RF models were retrained without SMOTETomek using three strategies: no class weighting, class_weight=“balanced”, and class_weight=“balanced_subsample”. Discrimination remained comparable to that of the optimized RF pipeline across all outcomes. The AUROCs of the no-resampling models ranged from 0.85 to 0.86 for bowel resection, 0.70 to 0.71 for perianal complications, and 0.73 to 0.74 for abdominal complications. Class weighting modestly improved sensitivity for perianal and abdominal complications, while calibration remained broadly comparable (**Supplementary Table 5**).

Based on its consistently strong overall performance across the three outcomes, RF was selected as the final model for subsequent interpretability and clinical-utility analyses.

### Different complications risk stratification ability of RF model

SHAP-based interpretation ranked disease behavior, particularly stricturing or penetrating patterns, as the strongest predictor of one-year bowel resection, followed by receipt of NST, disease duration, and age (**Figure 4A**). Patients receiving NST showed positive SHAP contributions and higher model-predicted probabilities of bowel resection. Because NST is commonly initiated in patients with nutritional depletion, impaired oral intake, intestinal obstruction, or greater disease severity, this pattern was interpreted as the model capturing the underlying depleted and clinically vulnerable state rather than an adverse effect of NST itself. Receipt of NST also contributed to the prediction of abdominal complications, although its contribution varied between individual patients.

Across complication subtypes, NST emerged as a consistent secondary contributor. In abdominal complications (task 3), it ranked immediately after prior complication history as a major predictor of elevated risk in high–risk individuals (Prob. = 0.512), while exerting negative contributions in low–risk patients (Prob. = 0.902). Conversely, perianal complications (task 2) were primarily driven by perianal disease history and clinical disease activity, with NST contributing modestly to risk discrimination. Collectively, SHAP visualizations highlight the stable role of NST in risk stratification for both bowel resection and abdominal complications within the one–year follow–up period. To illustrate how individual-level RF predictions could be displayed, we developed an exploratory nomogram and a web-based calculator based on the most influential features (**Figure 5**; **Supplementary Figures 2**, **3**). These visualizations summarize model-derived risk estimates but do not represent externally validated clinical decision aids.

### Associations of disease location phenotypes with distinct complication risks

Phenotypic stratification based on the Montreal classification revealed significant heterogeneity in disease activity and longitudinal complication trajectories. Patients with isolated colonic disease (Only–CI) exhibited significantly elevated CDAI scores compared to those with isolated small–bowel disease (Only–SI) or ileocolonic involvement (Others) (**Figure 6A**). Conversely, no significant disparity was found between the Only–SI and “Others” cohorts, implying a distinct association between colonic involvement and heightened clinical activity. These patterns were further substantiated in binary analyses (**Figure 6B**), where CDAI scores remained comparable between the Only–SI and consolidated non–isolated small–bowel groups.

Phenotype-stratified analyses included 93 patients with Only-SI, 47 with Only-CI, and 230 with other Montreal phenotypes (**Figures 6D–F**; **Supplementary Table 6**). For bowel resection, AUROCs were 0.812 (95% CI, 0.720–0.894), 0.874 (95% CI, 0.756–0.963), and 0.903 (95% CI, 0.844–0.950), respectively. The corresponding AUROCs were 0.743, 0.681, and 0.700 for perianal complications and 0.703, 0.659, and 0.764 for abdominal complications. Confidence intervals were wider in the smaller subgroups and overlapped substantially. Therefore, these results were interpreted as exploratory rather than evidence of differences in model performance between phenotypes.

Regarding one–year clinical outcomes (**Figure 6G**), the Only–SI group demonstrated a substantially higher incidence of bowel resection compared to the Only–CI and Others groups (34.4% vs. 12.8% vs. 13.9%). Perianal complications were most frequent among Only–CI patients (51.1%), whereas abdominal complications occurred more frequently within the Only–SI group (35.5%). These phenotypic associations remained robust in the binary analysis (**Figure 6H**); isolated small–bowel involvement (Only–SI) conferred a disproportionate risk for both bowel resection and abdominal complications, while non–isolated small–bowel phenotypes were characterized by a higher burden of perianal disease.

## Discussion

In this retrospective two-center study, we developed and evaluated an ML framework for predicting the one-year risk of three distinct complication categories in CD. By integrating pooled data from two clinical centers, interpretable RF modeling, and Montreal phenotype–based analyses, this study provides an exploratory framework for characterizing outcome-specific risk profiles in CD. By providing individualized prognostic estimates across a 12–month clinical trajectory, our approach transcends traditional population–level stratification. The identification of granular predictive factors through optimized algorithms enables IBD clinicians to shift from a reactive treatment paradigm toward proactive, early intensified therapy—a critical transition in the current “treat–to–target” era of IBD management.

Disease behavior, NST, and disease duration were among the leading model-associated predictors of bowel resection. Stricturing or penetrating behavior reflects established structural damage, whereas longer disease duration may capture accumulated bowel injury (Halawani et al., 2020; Liu et al., 2022). Additionally, longer disease duration further captures the cumulative burden of irreversible intestinal injury, leading to higher rates of disability and surgical requirements (Yasueda et al., 2022; Murthy et al., 2023). Furthermore, Receipt of NST during the index hospitalization was interpreted as a clinical proxy for nutritional depletion and greater disease burden (Meng et al., 2022). Patients requiring NST commonly have impaired nutritional intake, intestinal obstruction, advanced disease, or nutritional depletion and therefore represent a clinically vulnerable group with a less favorable prognosis and a higher model-predicted probability of bowel resection. Thus, our findings do not indicate that NST itself increases the risk of resection; rather, NST identifies patients whose underlying clinical condition has prompted nutritional intervention. SHAP values describe contributions to model predictions and do not establish causality.

Regarding perianal complications (task 2), SHAP analysis identified prior perianal history as the strongest risk contributor, reflecting the characteristically relapsing–remitting course of perianal CD (Schmidt et al., 2021; Brennan and Beniwal-Patel, 2025). In addition, the positive correlation of extra–intestinal manifestations and CDAI scores with higher SHAP values suggested that perianal outcomes were predominantly driven by the systematic inflammatory burden (Bao et al., 2022). In contrast to bowel resection, where disease behavior was the principal determinant, perianal complications appeared less influenced by the accrual of structural damage and more closely tethered to the magnitude of the active inflammatory burden. These observations underscore the necessity of early screening for high–risk patients to enable timely optimization of anti–inflammatory therapy and local perianal management to preserve long–term quality of life.

Regarding abdominal complications (Task 3), prior history emerged as the most significant predictor of recurrence within one year, highlighting the impact of pre–existing structural vulnerability and transmural disease behavior. According to the Montreal classification, lesion location demonstrated a clear gradient effect: ileocolonic involvement (L3) was associated with the highest risk (Torres et al., 2016; Pavel et al., 2023). Higher predicted risk was also associated with protracted disease duration, exacerbated abdominal pain, and poor general condition status, highlighting the synergistic impact of chronic inflammatory accrual and sustained symptom burden on abdominal disease progression (Panaccione et al., 2025).

As discussed above, the interpretable SHAP–RF framework identified divergent prognostic architectures across specific CD complications. Bowel resection (task 1) was primarily characterized by disease behavior and the accrual of structural damage, perianal complications (task 2) were more closely tethered to active inflammatory burden and prior perianal involvement, whereas abdominal complications (task 3) were most strongly predicted by anatomical disease extent and recurrence history.

In the conventional four-level Montreal analysis, disease location was not significantly associated with abdominal complications in the overall multivariable test. Therefore, the category-specific association observed for L3 relative to L1 should be regarded as exploratory. Although NST consistently emerged as a prominent contributor across both logistic regression (**Figure 2**) and SHAP–based analyses (**Figure 4**), it should not be interpreted as a causal risk factor for adverse outcomes. Rather, the initiation of NST likely reflects underlying disease severity, nutritional depletion, or structural complications that necessitate intensified supportive therapy. In this context, NST functions as a surrogate marker of high–risk clinical states rather than a direct driver of bowel resection or abdominal complications. The reproducible identification of NST across models underscores the strength of interpretable machine learning in capturing real–world treatment–severity coupling embedded within clinical decision–making, reinforcing its value for phenotype–aware risk stratification and early identification of patients requiring proactive, intensified intervention. Therefore, the model should be interpreted as a discharge-time prognostic tool and not as a pretreatment or admission-time prediction model. More broadly, all variables identified by regression and SHAP were interpreted as prognostic markers rather than causal risk factors.

Previous ML studies in IBD have addressed treatment response, disease activity, diagnosis, relapse, postoperative outcomes, and other prognostic endpoints using clinical, laboratory, imaging, or multimodal data (Gomollón et al., 2022; Guez et al., 2022; Hugo et al., 2023; Wang et al., 2025; Xiang et al., 2025; Xia et al., 2025). Systematic reviews nevertheless indicate that many published models have limited external validation and remain at substantial risk of bias (Nguyen et al., 2022; Da Rio et al., 2023). The present study should therefore be viewed as an extension of this literature rather than the first interpretable ML framework in IBD. Its main contribution is the evaluation of three one-year outcomes within a common RF–SHAP framework, together with exploratory assessment across Montreal phenotypes.

Compared with the study by Xia et al (Xia et al., 2025), firstly, their study previously targeted clinical remission during Adalimumab therapy at 12 and 48 weeks, whereas the present study specifically predicts a spectrum of CD–related complications over a one–year post–discharge trajectory, which acquired different results; secondly, our cohort included patients from two clinical centers, increasing the clinical heterogeneity of the pooled dataset, although independent center-wise external validation was not performed; thirdly, we integrated a more comprehensive panel of systemic inflammatory biomarkers and demographic variables to reflect the holistic disease status of patients with CD; and fourthly, a targeted prognostic analysis stratified by specific phenotypes to address the impact of anatomical heterogeneity on complication risks were performed in our present study. The present study extends previous ML research in IBD by combining SHAP-based interpretation and visual risk-estimation tools for three clinically distinct one-year outcomes within a common analytical framework, providing transparent and exploratory visualizations of model-derived risk. However, their transportability and clinical usefulness remain to be established through independent external validation.

Notably, although patients with isolated small–bowel involvement had lower mean CDAI scores than those with isolated colonic disease (**Figure 6B**), they showed a higher risk of subsequent bowel resection and abdominal complications (**Figures 6J, L**). However, CDAI scores were significantly and consistently associated with higher risks of all three categories in an approximately linear manner, indicating that greater disease activity confers an increased likelihood of complications (**Figures 6D–F**). This discordance indicates a dissociation between symptom–based CDAI assessment and long–term adverse outcomes in small–bowel CD, likely reflecting under–recognition of occult inflammation or structural progression. Consistently, phenotype–stratified ROC analyses revealed substantial heterogeneity in predictive discrimination, with attenuated performance in isolated small–bowel disease compared with mixed–involvement phenotypes (**Figures 6G–I**). Together, these findings highlight the limitations of symptom–driven indices and underscore the need for phenotype–aware, outcome–oriented risk stratification.

Our explainable findings further support a paradigm of phenotype–informed risk stratification, thereby emphasizing the necessity of early identification and the timely implementation of targeted management strategies. To halt disease progression, clinical focus must be meticulously tailored to specific risk profiles: patients with these characteristics showed higher observed event rates, while perianal complications demand intensified surveillance and integrated surgical management for patients, in whom risk is concentrated among those with a history of perianal disease, extra–intestinal manifestations, and elevated CDAI scores. Furthermore, vigilance regarding disease extent (particularly L3) and recurrence remains pivotal to reducing abdominal complications and improving long–term quality of life. It is important that these findings should be regarded as hypothesis-generating and should not be interpreted as model-based treatment recommendations. The clinical consequences of model-guided surveillance or treatment escalation require prospective evaluation.

In this study, the optimized RF model demonstrated the most consistent overall performance across the three outcome categories (**Table 2**, **Figure 3**). PR curves for the RF model exhibited a favorable precision–recall profile, indicating enhanced robustness in the context of imbalanced clinical datasets. DCA further confirmed the potential clinical utility, showing a higher net benefit over selected threshold ranges, while calibration curves indicated reasonable agreement between predicted and observed risks. These favorable outcomes likely arise from the ensemble decision architecture of RF, which minimizes generalization error by aggregating diverse, decorrelated decision trees built through bootstrap resampling. Specifically, the RF model effectively captures heterogeneous CD phenotypes through adaptive non–linear splits, while its voting–based probability smoothing stabilizes the detection of low–prevalence complications, supporting further evaluation for potential clinical application.

Although the internally cross-validated AUROCs for perianal and abdominal complications were 0.71 and 0.73, respectively, and were lower than the AUROC of 0.86 for bowel resection, the models retained moderate discrimination within the pooled two-center cohort. Importantly, the recall and specificity estimates varied across outcomes, with the strongest overall performance observed for bowel resection. This study extends previous ML research in IBD by evaluating three distinct one-year outcomes and integrating SHAP-based interpretation with exploratory Montreal phenotype–stratified analyses.

The comparable performance of the no-resampling models suggests that the main findings were not dependent on synthetic oversampling. In this cohort, class weighting provided a simpler alternative while preserving overall discrimination and calibration.

This study has several limitations. Firstly, although patients were recruited from two clinical centers, data from both centers were pooled before cross-validation; therefore, the analysis did not constitute independent center-wise external validation. Secondly, the sample size was modest, particularly for phenotype-specific subgroup analyses, and the cohort was geographically concentrated in southern China. Thirdly, the absence of imaging–derived features, such as endoscopic or radiological assessments, may constrain the model’s ability to capture morphology–driven risk signatures. Fourthly, because NST is treatment-related, the model cannot distinguish the effect of the treatment itself from the underlying disease state that prompted its use. Finally, the nomograms and web-based calculator should be regarded as exploratory research prototypes and have not yet been implemented within hospital information systems (HIS) (Chen et al., 2025), and its clinical impact therefore remains to be prospectively evaluated.

Future research should prioritize large-scale, prospective multicenter validation to evaluate model transportability and calibration across diverse CD populations and clinical settings. The integration of multimodal data, including radiological imaging, endoscopic indices, and emerging molecular profiles, may further enhance predictive granularity and biological interpretability. Beyond model refinement, embedding interpretable ML tools into web–based or HIS could enable real–time, phenotype–aware risk stratification in routine care. Ultimately, the development of an AI–driven foundation model that integrates longitudinal disease trajectories with large language model–augmented clinical knowledge represents a promising direction for advancing proactive, personalized management and improving long–term outcomes in Crohn’s disease.

In conclusion, we developed an interpretable ML framework for predicting one–year complication risk in CD. RF showed promising cross-validated discrimination, particularly for bowel resection, while performance for perianal and abdominal complications was more moderate. The nomograms and web-based calculator remain exploratory. Larger independent cohorts are required to evaluate model transportability, calibration, and potential clinical utility.

## Data Availability

The raw data supporting the conclusions of this article will be made available by the authors, without undue reservation.
