# Correction: Explainable random forest–SHAP framework for Montreal phenotype–stratified prediction of one-year complications in Crohn’s disease

**DOI:** 10.3389/fphys.2026.2018653

**Published:** 2026-10-09

**Authors:** Kun Xia, Ying Shi, Xiaohan Huang, Yuwei Zhang, Liwei Suo, Shuntian Cai, Lingfeng Zhong, Dayi Zheng, Mengjie Lin, Sirui Wan, Linwei Wang, Jianlin Ren, Qingan Xia, Hongzhi Xu, Yanyun Fan

**Affiliations:** 1 Department of Gastroenterology, The National Key Clinical Specialty, Zhongshan Hospital of Xiamen University, School of Medicine, Xiamen University, Xiamen, Fujian, China; 2 Department of Health Technology and Informatics, The Hong Kong Polytechnic University, Hong Kong, Hong Kong SAR, China; 3 Clinical Research Center for Gut Microbiota and Digestive Diseases of Fujian Province, Xiamen Key Laboratory of Intestinal Microbiome and Human Health, Xiamen, Fujian, China; 4 Department of Digestive Disease, Institute for Microbial Ecology, Xiamen University, Xiamen, Fujian, China; 5 Gastroenterology Department, First Affiliated Hospital of Jinan University, Guangzhou, Guangdong, China; 6 Department of Infectious Diseases, The Second Affiliated Hospital of Xiamen Medical College, Xiamen, Fujian, China; 7 Xiamen Gastroenterology Quality Control Center, Xiamen, Fujian, China; 8 Department of Pathology, Zhongshan Hospital of Xiamen University, School of Medicine, Xiamen University, Xiamen, Fujian, China; 9 Hospital Clínic–IDIBAPS, University of Barcelona, Barcelona, Spain; 10 Department of Pathology, TangShan GongRen Hospital, Tangshan, Hebei, China

**Keywords:** artificial intelligence, Crohn’ s disease, inflammatory bowel disease, machine learning, prognosis factors

In the published article, affiliation 6 “Department of Infectious Diseases, The Second Affiliated Hospital of Xiamen Medical College, Xiamen, Fujian, China” was omitted. This affiliation has now been added for author “Xiaohan Huang”.

The original version of this article has been updated.

