# Supplementary material for: Explainable random forest–SHAP framework for Montreal phenotype–stratified prediction of one-year complications in Crohn’s disease
Source: Front Physiol. 2026 Sep 15;17:1902465. doi: 10.3389/fphys.2026.1902465 (PMC13619521; doi:10.3389/fphys.2026.1902465)
Supplement: Supplementary file 1 [file DataSheet1.pdf]

Supplementary Materials Contents

1、 **Supplementary Methods S1**--002–003

2、 **Figure S1. Exploratory visualization (top 10 most influential variables identified by SHAP) of model-derived risk estimates for perianal complications (Task 2).** ----------------------004

3、 **Figure S2. Exploratory visualization (top 10 most influential variables identified by SHAP) of model-derived risk estimates for abdominal complications (Task 3).** --------------------005

4、 **Figure S3. Additional model performance evaluation for Tasks 1–3 in the test cohort (mean of 10-fold cross-validation).** ---006

5、 **Figure S4. Joint plots for correlation between various laboratory test metrics and clinical disease activity (CDAI score).** --007

6、 **Figure S5. Partial dependence plots (PDPs) for the top four features highest-ranked by mean absolute SHAP values in each task.** --008

7、 **Table S1: Comparison of baseline demographic and clinical characteristics between patients with and without bowel resection complications (BRC).** --------------------009–011

8、 **Table S2: Comparison of baseline demographic and clinical characteristics between patients with and without perianal complications (ANC).** ------------------------------012–014

9、 **Table S3: Comparison of baseline demographic and clinical characteristics between patients with and without abdominal complications (ABC).** --------------------------015–017

10、 **Table S4. Missingness and analytical inclusion status of candidate variables.** ------018-019

11、 **Table S5. Ablation analysis of SMOTETomek and class-weighting strategies in the Random Forest models.** ---020

12、 **Table S6. Montreal phenotype–stratified sample sizes, event frequencies, complication rates, and model discrimination.** ---021

Supplementary Methods S1

Complete model-development procedure

Missing-data assessment

Missingness was assessed before model fitting and is reported for each variable in Supplementary Table S4. Variables with ≥20% missingness were excluded: ustekinumab (82.50%), infliximab treatment category (29.69%), inadequate response (24.06%), infliximab dose escalation (23.13%), infliximab interval shortening (23.13%), azathioprine (21.57%), adalimumab (21.25%), and mesalazine (20.00%). Missingness among retained variables ranged from 0.31% to 13.75%; all other variables were complete.

Nested cross-validation and LASSO selection

Model performance was estimated using nested, stratified 10-fold cross-validation with shuffling and random state 42. In each outer iteration, 90% of patients formed the outer-training set and 10% formed an untouched outer-test set. Standardization, feature selection, resampling, model tuning, calibration, and threshold selection were restricted to the outer-training data.

LASSO selection used L1-penalized logistic regression with the liblinear solver and a maximum of 1,000 iterations. The penalty was selected by stratified five-fold cross-validation from 100 logarithmically spaced values of λ between 10^{-3} and 10^{1} ($C = 1/\lambda$), using mean validation AUROC. Predictors with non-zero coefficients were retained. During RF tuning, the selected C and up to four adjacent values on either side were re-evaluated, with the selector refitted in every inner-training partition.

$$\beta(\lambda) = \arg\min_{\beta} \left[-\ell(\beta; X, y) + \lambda \sum_{j=1}^{p} |\beta_j| \right] \quad (1)$$

where ℓ denotes the logistic log-likelihood and λ controls the degree of coefficient shrinkage.

Random Forest optimization and SMOTE-Tomek

Random Forest (RF) models used bootstrap sampling, class_weight="balanced_subsample", oob_score=True, and random state 42. Sixty parameter combinations were evaluated using RandomizedSearchCV with stratified five-fold inner cross-validation and AUROC as the optimization metric.

- **Bowel resection:** n_estimators={200, 500, 800, 1,200}; max_depth={8, 10, 12, 15, None}; max_features={sqrt, log2, 0.3, 0.5}; min_samples_split={5, 10, 15, 20, 25}; min_samples_leaf={1, 2, 4, 6, 8}.
- **Perianal complications:** n_estimators={500, 800, 1,200}; max_depth={8, 10, 12, 15, 20, None}; max_features={sqrt, log2, 0.3, 0.5, 0.7}; min_samples_split={2, 5, 10, 15, 20}; min_samples_leaf={1, 2, 3, 4, 5, 8}.

- **Abdominal complications:** n_estimators={300, 500, 800, 1,200}; max_depth={6, 8, 10, 12, 15, None}; max_features={sqrt, log2, 0.3, 0.5, 0.7}; min_samples_split={2, 5, 10, 15, 20, 25}; min_samples_leaf={1, 2, 3, 4, 6, 8}.

For all tasks, criterion={gini, entropy, log_loss} and max_samples={0.6, 0.7, 0.8, 1.0}. SMOTE-Tomek was specified as SMOTETomek(sampling_strategy=1.0, random_state=42) after standardization and LASSO selection and was compared with no resampling (passthrough) during inner tuning. Synthetic samples were generated only from training-partition observations:

$$x_{\text{new}} = x_i + \delta(x_{\text{NN}} - x_i), \delta \sim \text{U}(0,1) \quad (2)$$

SMOTE-Tomek was selected in 6/10 bowel-resection folds, 8/10 perianal-complication folds, and 2/10 abdominal-complication folds. The sampler was never fitted to or applied to an outer-test fold.

Calibration and decision-threshold selection

The selected pipeline was calibrated using sigmoid calibration with stratified three-fold cross-validation, random state 1042, and ensemble=False. The calibrated probability was:

$$\hat{p}_{\text{cal}}(x) = \frac{1}{1+\exp\{Af(x)+B\}} \quad (3)$$

A fold-specific threshold was derived from five-fold out-of-fold probabilities generated within the outer-training set by maximizing Youden's J statistic:

$$t^* = \arg\max_t[\text{Sensitivity}(t) + \text{Specificity}(t) - 1] = \arg\max_t[\text{TPR}(t) - \text{FPR}(t)] \quad (4)$$

The fitted pipeline, sigmoid calibrator, and training-derived threshold were then applied unchanged to the corresponding outer-test fold.

Fold-specific selected features and final settings

Because LASSO selection and RF optimization were repeated within each outer fold, there was no single feature set or parameter vector shared by all held-out patients. Features retained in all 10 folds were: bowel resection—age, sex, disease duration, disease behavior, lesion location (L1/L2/L3), vomiting, total enteral nutrition, abdominal-complication history, and other-organ-complication history; perianal complications—abdominal-complication history, perianal-complication history, diarrhea, extramural complications, folic acid, gastrointestinal bleeding, total enteral nutrition, and total iron-binding capacity; abdominal complications—abdominal-complication history, lesion location (L1/L2/L3), and total enteral nutrition.

A

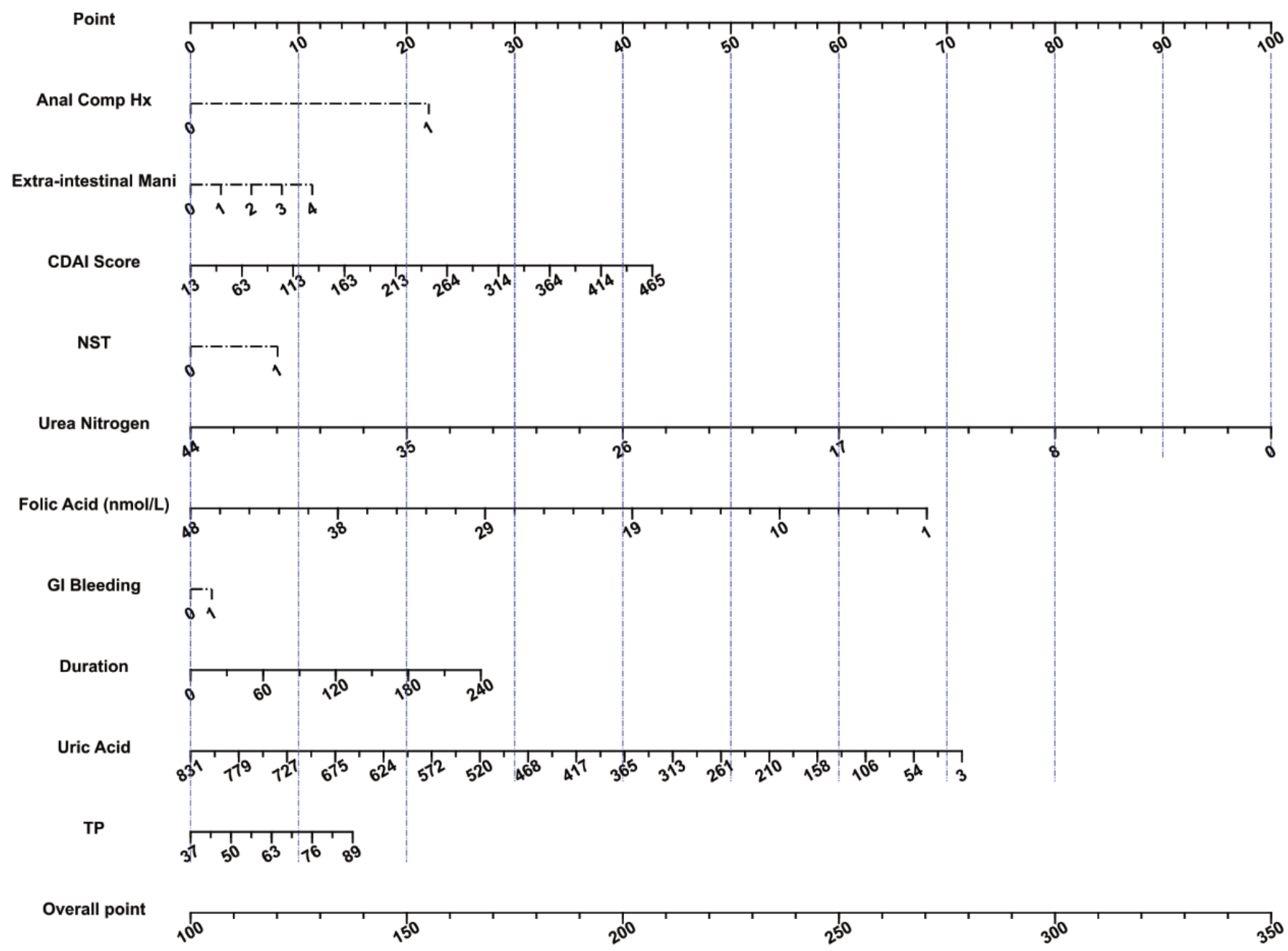

B

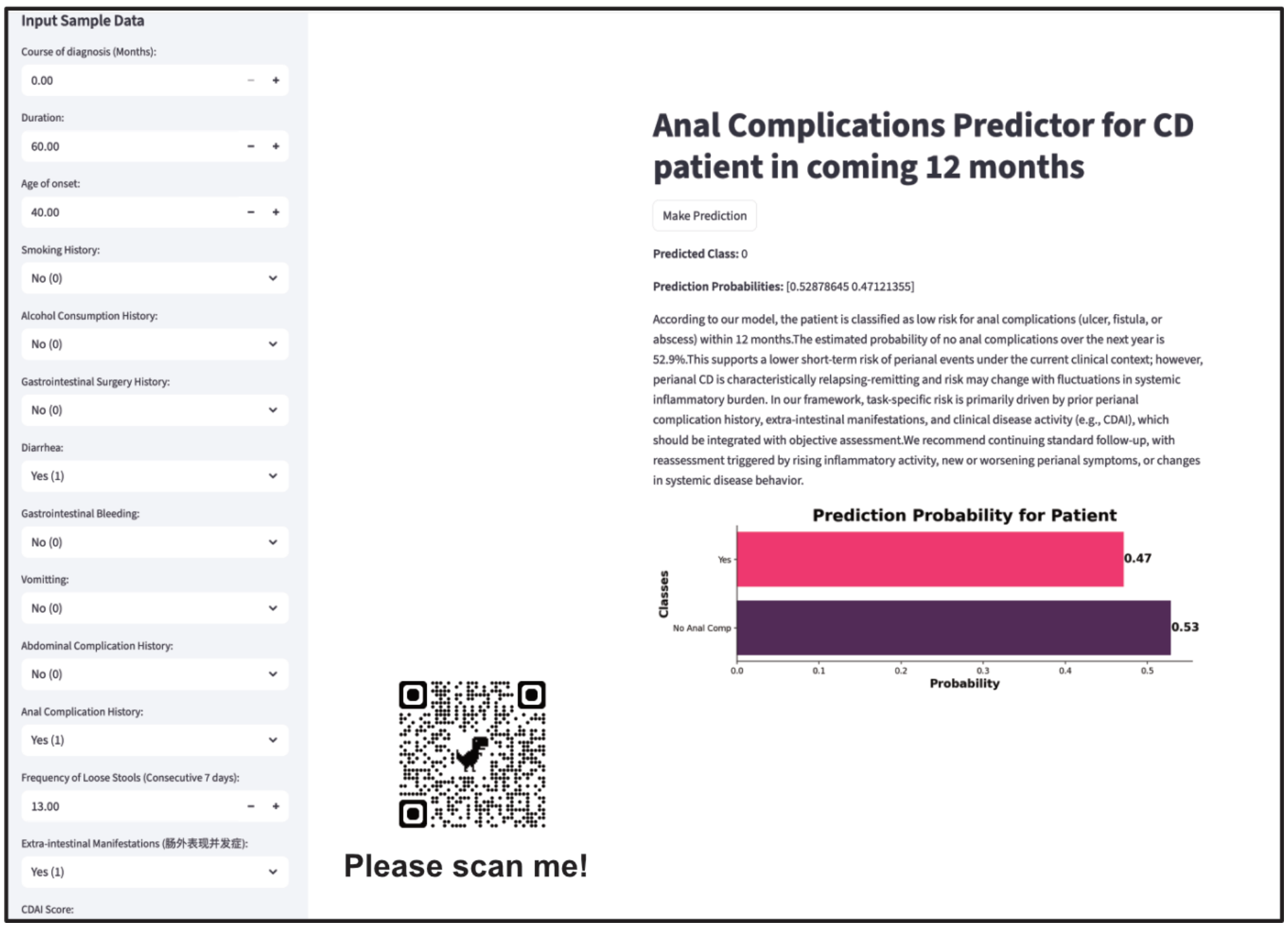

Figure S1. Exploratory visualization (top 10 most influential variables identified by SHAP) of model-derived risk estimates for perianal complications (Task 2). A quantified score is assigned to each prognostic factor on the points scale. The total points, calculated by summing the scores from all factors, are subsequently translated into an individual estimate of positive risk. Anal Comp Hx, anal complications history. Extra Comp, extraintestinal complications. TP, total protein. NST, nutritional support therapy. GI Bleeding, gastrointestinal bleeding.

A

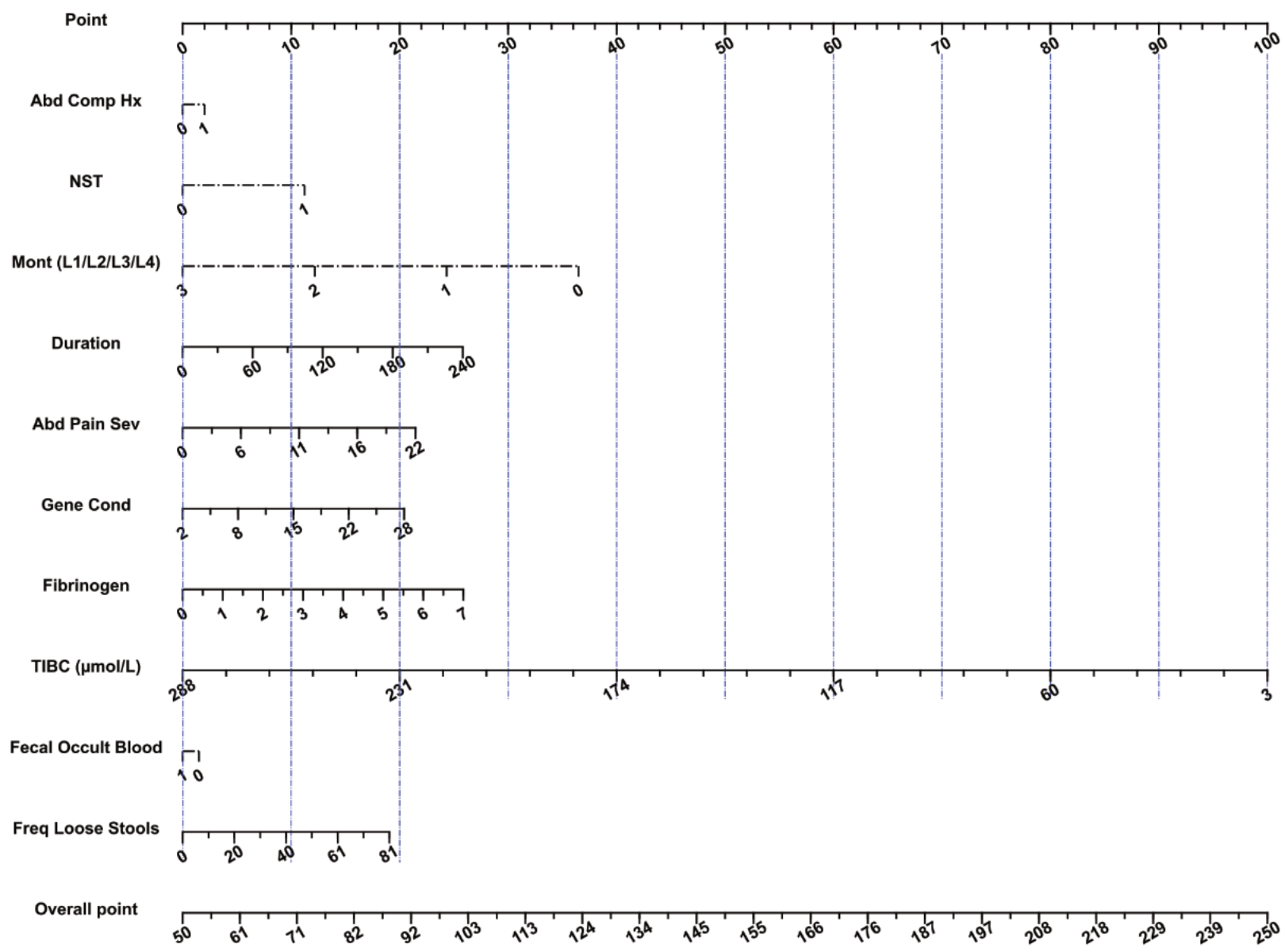

B

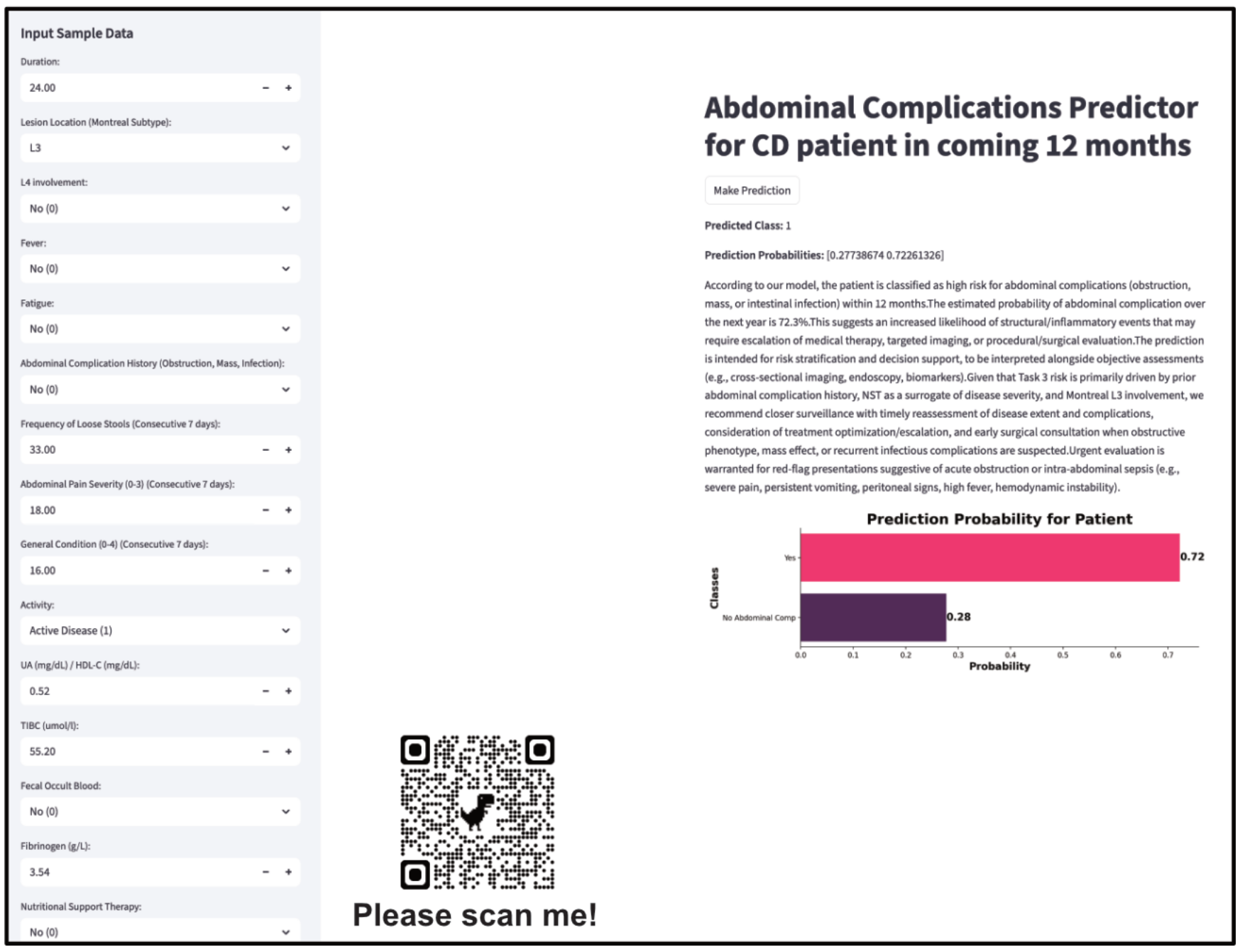

Figure S2. Exploratory visualization (top 10 most influential variables identified by SHAP) of model-derived risk estimates for abdominal complications (Task 3). A quantified score is assigned to each prognostic factor on the points scale. The total points, calculated by summing the scores from all factors, are subsequently translated into an individual estimate of positive risk. Abd Comp Hx, abdominal complications history. TIBC (μmol/L), total iron-binding capacity (μmol/l). Abd Pain Sev, abdominal Pain Severity (0-3). Gene Cond, general condition (0-4). Freq Loose Stools, frequency of loose stools. NST, nutritional support therapy.

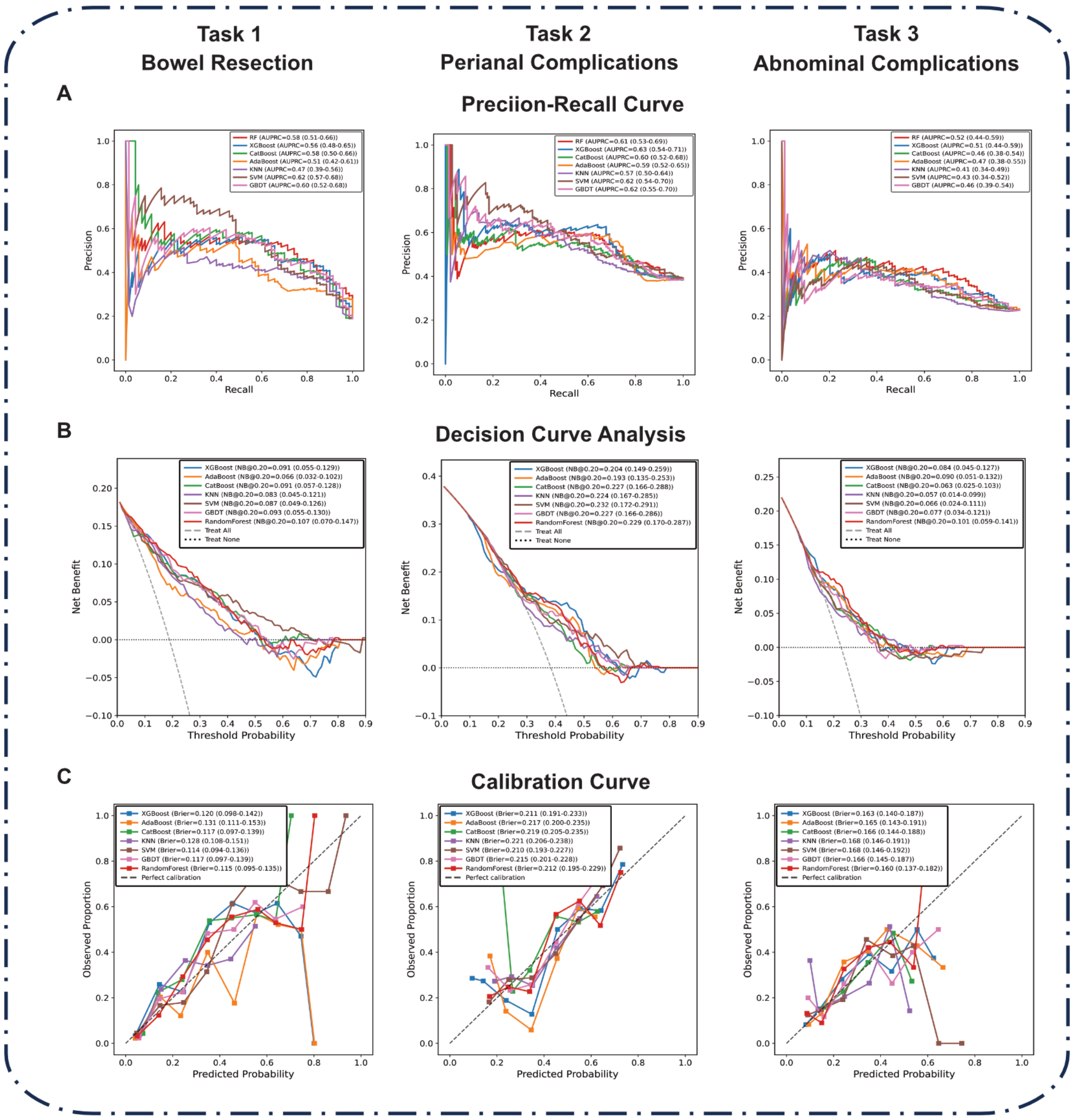

Figure S3. Additional model performance evaluation for Tasks 1–3 in the test cohort (mean of 10-fold cross-validation). (A) Precision–Recall curves. (B) Decision Curve Analysis. (C) Calibration Curves.

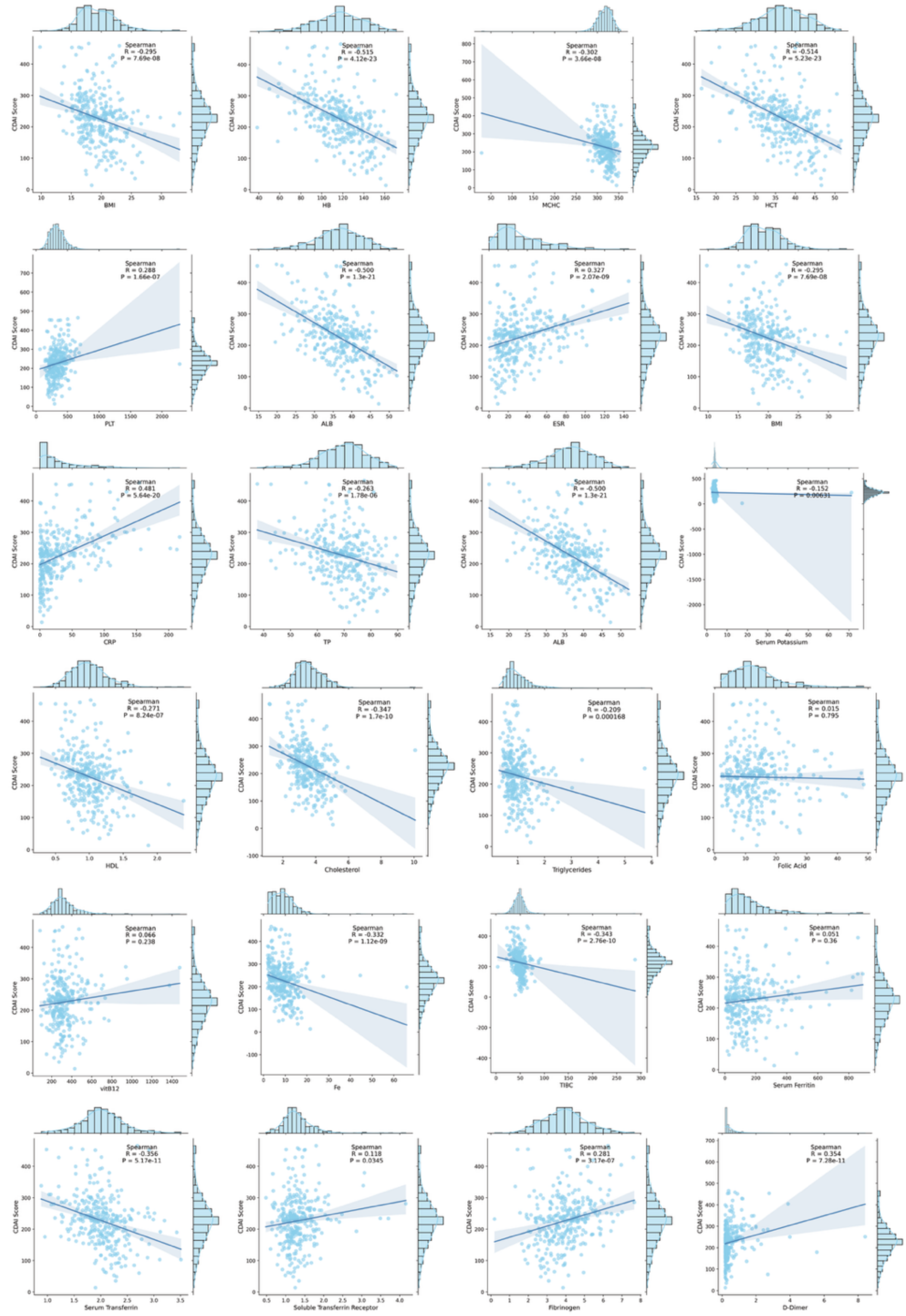

Figure S4. Joint plots for correlation between various laboratory test metrics and clinical disease activity (CDAI score). These joint plots illustrate the bivariate relationships between candidate laboratory biomarkers (x-axis) and CDAI scores (y-axis), and display bivariate scatter with regression lines (95% CI) and marginal densities. Spearman's "R" quantifies the monotonic association, with "P" denoting statistical significance.

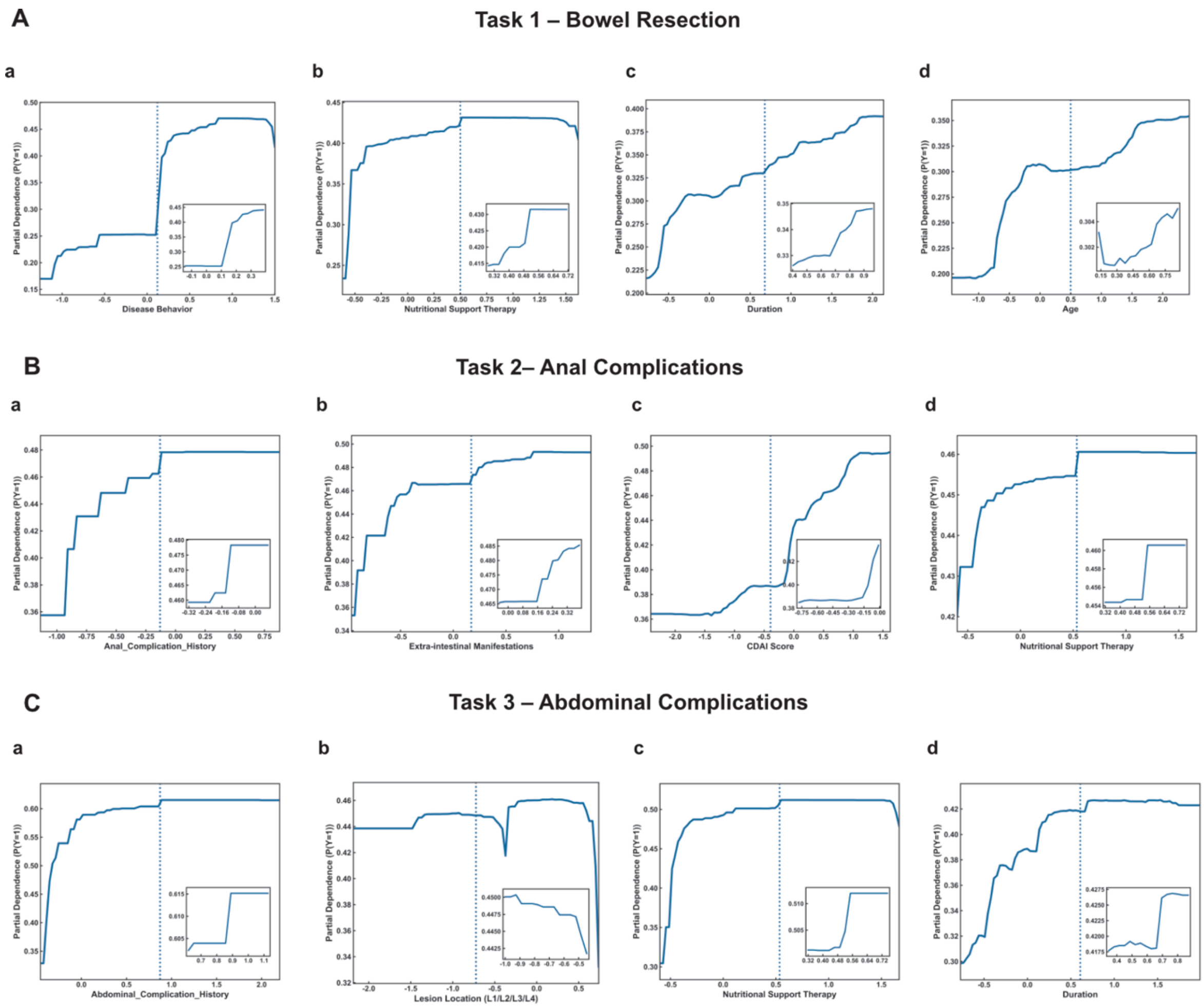

Figure S5. Partial dependence plots (PDPs) for the top four features highest-ranked by mean absolute SHAP values in each task. For each panel, the y-axis indicates the model-estimated partial dependence (predicted probability of the outcome) while varying the feature of interest and marginalizing over the remaining covariates. The dashed vertical line denotes the reference value (median) of the corresponding feature. Insets provide a zoomed view around the central range to facilitate visualization of subtle changes.

Variable	Total (N = 370)	No BRC (N = 300)	BRC (N = 70)	χ2 /Z	P
Sex, n (%)					
Male	102 (27.6)	87 (29.0)	15 (21.4)	1.629[a]	0.202
Female	268 (72.4)	213 (71.0)	55 (78.6)		
Age, (Years)	28.0 (22, 35.2)	27.0 (21.0, 34.0)	31.0 (28, 43.2)	-4.173[b]	< 0.001*
Course of diagnosis, (Months)	0.0 (0.0, 9.0)	0.0 (0.0, 4.0)	9.0 (0.0, 48.0)	-3.937[b]	＜0.001*
Symptoms to diagnosis, (Months)	6.0 (0.5, 24.0)	6.0 (1.0, 15.5)	12.0 (0.0, 36.0)	-1.202[b]	0.229
Duration, (Months)	12.0 (4.0, 45.0)	12.0 (4.0, 36.0)	43.0 (12.0, 96.0)	-5.233[b]	＜0.001*
Age of onset, (Years)	26.0 (20.0, 33.0)	26.0 (20.0, 33.0)	28.0 (23.0, 39.5)	-2.805[b]	0.005*
BMI, (kg/m^2)	19.0 (17.3, 21.5)	19.2 (17.3, 21.6)	18.5 (16.9, 20.8)	-1.686[b]	0.092
Smoking History, n (%)					
No	323 (87.3)	266 (88.7)	57 (81.4)	2.681[a]	0.102
Yes	47 (12.7)	34 (11.3)	13 (18.6)		
Alcohol Consumption History, n (%)					
No	340 (91.9)	278 (92.7)	62 (88.6)	1.278[a]	0.258
Yes	30 (8.1)	22 (7.3)	8 (11.4)		
Hepatitis B, n (%)					
No	333 (90.0)	270 (90.0)	63 (90.0)	＜0.001[a]	1.000
Yes	37 (10.0)	30 (10.0)	7 (10.0)		
Abdominal History, n (%)					
No	192 (51.9)	166 (55.3)	26 (37.1)	7.523[a]	0.006*
Yes	178 (48.1)	134 (44.7)	44 (62.9)		
Disease Behavior, n (%)					
No-stricturing & No-penetrating	113 (30.5)	112 (37.3)	1 (1.4)	47.075[a]	＜0.001*
Stricturing	173 (46.8)	137 (45.7)	36 (51.4)		
Penetrating	84 (22.7)	51 (17)	33 (47.2)		
Lesion Location, n (%)					
L1	55 (14.9)	34 (11.3)	21 (30.0)	19.696[a]	0.001*
L2	47 (12.7)	41 (13.7)	6 (8.6)		
L3	137 (37.0)	121 (40.3)	16 (22.9)		
L4	7 (1.9)	5 (1.7)	2 (2.9)		
Merge, n (%)	124 (33.5)	99 (33.0)	25 (35.7)		
Diarrhea, n (%)					
No	103 (27.8)	85 (28.3)	18 (25.7)	0.194[a]	0.660
Yes	267 (72.2)	215 (71.7)	52 (74.3)		
Abdominal Pain, n (%)					
No	57 (15.4)	52 (17.3)	5 (7.1)	4.523[a]	0.033*
Yes	313 (84.6)	248 (82.7)	65 (92.9)		
Abdominal Bloating, n (%)					
No	282 (76.2)	237 (79)	45 (64.3)	6.779[a]	0.009*
Yes	88 (23.8)	63 (21.0)	25 (35.7)		
Gastrointestinal Bleeding, n (%)					
No	255 (68.9)	202 (67.3)	53 (75.7)	1.861[a]	0.172
Yes	115 (31.1)	98 (32.7)	17 (24.3)		
Weight Loss, n (%)					
No	218 (58.9)	177 (59.0)	41 (58.6)	0.004[a]	0.948
Yes	152 (41.1)	123 (41.0)	29 (41.4)		
Vomiting, n (%)					
No	321 (86.8)	275 (91.7)	46 (65.7)	33.272[a]	＜0.001*
Yes	49 (13.2)	25 (8.3)	24 (34.3)		
Nausea, n (%)					
No	312 (84.3)	268 (89.3)	44 (62.9)	30.099[a]	＜0.001*
Yes	58 (15.7)	32 (10.7)	26 (37.1)		

Variable	Total (N = 370)	No BRC (N = 300)	BRC (N = 70)	χ2 /Z	P
Fever, n (%)					
No	302 (81.6)	246 (82.0)	56 (80.0)	0.151[a]	0.697
Yes	68 (18.4)	54 (18.0)	14.0 (20.0)		
Anemia, n (%)					
None	160 (43.2)	137 (45.7)	23 (32.9)	-1.595[b]	0.111
Mild	155 (41.9)	119 (39.7)	36 (51.4)		
Moderate	50 (13.5)	39 (13.0)	11 (15.7)		
Severe	5 (1.4)	5 (1.7)	0 (0.0)		
Poor Appetite, n (%)					
No	273 (73.8)	225 (75)	48 (68.6)	1.213[a]	0.271
Yes	97 (26.2)	75 (25)	22 (31.4)		
Fatigue, n (%)					
No	303 (81.9)	249 (83.0)	54 (77.1)	1.313[a]	0.252
Yes	67 (18.1)	51 (17.0)	16 (22.9)		
Another Organ Complication History, n (%)					
No	327 (88.4)	269 (89.7)	58 (82.9)	2.562[a]	0.109
Yes	43 (11.6)	31 (10.3)	12 (17.1)		
Abdominal Complication History, n (%)					
No	308 (83.2)	267 (89.0)	41 (58.6)	37.674[a]	＜0.001*
Yes	62 (16.8)	33 (11.0)	29 (41.4)		
Anal Complication History, n (%)					
No	162 (43.8)	131 (43.7)	31 (44.3)	0.009[a]	0.925
Yes	208 (56.2)	169 (56.3)	39 (55.7)		
Frequency of Loose Stools	10.0 (0.0, 21.0)	11.5 (0.0, 21.0)	8.5 (0.0, 16.5)	-0.879[b]	0.379
Abdominal Pain Severity (0-3)	7.0 (2.0, 14.0)	7.0 (1.0, 14.0)	10.0 (7.0, 14.2)	-2.559[b]	0.011*
General Condition (0-4)	14.0 (7.0, 17.0)	14.0 (7.0, 16.8)	14.0 (10.0, 19.2)	-2.543[b]	0.011*
Extramural Complications, n (%)					
0	155 (41.9)	136 (45.3)	19 (27.1)	-2.487[a]	0.013*
1	130 (35.1)	98 (32.7)	32 (45.7)		
2	72 (19.5)	58 (19.3)	14 (20.0)		
3	11 (3.0)	7 (2.3)	4 (5.7)		
4	2 (0.5)	1 (0.3)	1 (1.4)		
Opioid Antidiarrheal Medications, n (%)					
No	369 (99.7)	299 (99.7)	70 (100.0)	0.234[a]	1.000
Yes	1 (0.3)	1 (0.3)	0 (0.0)		
Reduced Hematocrit	2.0 (0, 5.9)	1.7 (0.0, 5.7)	3.7 (0.0, 7.3)	-2.487[b]	0.013*
(Standard - Actual) / Standard Weight	13.0 (2.9, 22.2)	12.2 (2.1, 21.8)	16.4 (5.5, 23.1)	-1.620[b]	0.105
CDAI Score	222.8 (153.3, 260.2)	204.0 (138.5, 254.9)	235.8 (188.1, 285.4)	-3.355[b]	0.001*
Activity, n (%)					
Remission	87 (23.5)	78 (26.0)	9 (12.9)	5.451[a]	0.020*
Active	283 (76.5)	222 (74.0)	61 (87.1)		
WBC (×10^9)	6.6 (5.2, 8.7)	6.6 (5.4, 8.7)	5.6 (4.6, 8.6)	-1.827[b]	0.068
RBC (×10^12)	4.5 (4.0, 5.0)	4.5 (4.1, 5.0)	4.3 (3.9, 5.0)	-1.836[b]	0.066
HB (g/L)	117.5 (102.0, 133.0)	118.5 (103.0, 134.8)	111.0 (95.0, 125.2)	-2.260[b]	0.024*
MCV (fL)	83.9 (78.0, 88.0)	83.9 (78.8, 87.9)	83.3 (76.2, 88.8)	-0.091[b]	0.928
MCH (Pg)	26.9 (24.2, 28.5)	26.9 (24.2, 28.5)	26.2 (23.9, 28.7)	-0.358[b]	0.720
MCHC (g/L)	318.4 (307.0, 329.0)	318.4 (307.2, 329.0)	318.5 (307.0, 328.8)	-0.108[b]	0.914
HCT (%)	36.9 (32.8, 40.9)	37.2 (33.2, 41.5)	36.0 (31.8, 39.5)	-2.110[b]	0.035*
PLT (×10^9)	325.5 (256.8, 404.0)	333.0 (261.8, 414.0)	304.0 (240.8, 372.0)	-2.284[b]	0.022*
ESR (mm/h)	26.6 (15.8, 47.7)	27.8 (16.4, 48.7)	24.2 (12.9, 43.7)	-1.621[b]	0.105
CRP (mg/L)	17.1 (5.0, 51.1)	17.1 (4.9, 50.4)	19.6 (6.2, 53.0)	-0.441[b]	0.660

Variable	Total (N = 370)	No BRC (N = 300)	BRC (N = 70)	χ2 /Z	P
TP (g/L)	69.7 (63.2,75.1)	70.0 (63.9,75.2)	68.2 (59.6,72.8)	-2.176[b]	0.030*
ALB (g/L)	37.0 (32.9, 40.6)	37.2 (33.0, 40.7)	35.0 (32.1, 40.2)	-1.705[b]	0.088
Urea Nitrogen (mmol/L)	3.7 (3.0, 4.6)	3.7 (3.0, 4.6)	3.9 (3.2, 4.6)	-0.935[b]	0.350
Serum Potassium (mmol/L)	3.9 (3.6, 4.2)	3.9 (3.7, 4.2)	3.9 (3.6, 4.2)	-0.387[b]	0.699
Uric Acid (μmol/L)	333.8 (266.5, 393.5)	342.0 (276.6, 398.0)	312.5 (251.1, 350.6)	-3.167[b]	0.002*
HDL (mmol/L)	1.0 (0.8, 1.1)	1.0 (0.8, 1.2)	0.9 (0.8, 1.1)	-1.198[b]	0.231
Cholesterol (mmol/L)	3.5 (3.0, 4.1)	3.6 (3.0, 4.1)	3.3 (3.0, 4.0)	-1.870[b]	0.062
Triglycerides (mmol/L)	0.9 (0.7, 1.2)	0.9 (0.7, 1.2)	0.9 (0.7, 1.2)	-0.313[b]	0.754
Folic Acid (nmol/L)	11.5 (7.6, 16.2)	11.1 (7.5, 15.5)	14.2 (8.1, 18.5)	-1.850[b]	0.064
VitB12 (pmmol/L)	310.7 (260.7, 384.8)	316.4 (269.1, 384.7)	284.0 (228.2, 387.5)	-1.844[b]	0.065
Fe (umol/L)	8.4 (5.2, 11.8)	8.4 (5.2, 12.0)	8.8 (4.7, 11)	-0.974[b]	0.330
Total Iron-Binding Capacity (umol/L)	48.9 (42.0, 54.4)	49.5 (42.7, 55.2)	46.8 (39.6, 51.0)	-2.851b	0.004*
Serum Ferritin (ng/mL)	128.0 (66.5, 205.1)	135.1 (65.2, 210.4)	120.2 (69.8, 195.0)	-0.582[b]	0.561
Serum Transferrin (g/L)	2.0 (1.8, 2.2)	2.0 (1.8, 2.3)	2.0 (1.7, 2.1)	-1.842[b]	0.065*
Soluble Transferrin Receptor (mg/L)	1.3 (1.1, 1.6)	1.3 (1.1, 1.7)	1.3 (1.1, 1.5)	-0.964[b]	0.335
Fecal Occult Blood, n (%)					
No	74 (20.0)	60 (20.0)	14 (20.0)	＜0.001[a]	1.000
Yes	296 (80.0)	240 (80.0)	56 (80.0)		
Calprotectin, n (%)					
No	11 (3.0)	9 (3.0)	2 (2.9)	0.004[a]	1.000
Yes	359 (97.0)	291 (97.0)	68 (97.1)		
Fibrinogen (mg/L)	4.0 (3.2, 4.7)	4.0 (3.3, 4.8)	3.9 (2.5, 4.6)	-2.031[b]	0.042*
D-Dimer (mg/L)	0.3 (0.2, 0.6)	0.3 (0.2, 0.54)	0.4 (0.2, 0.8)	-2.111[b]	0.035*
Total Enteral Nutrition, n (%)					
No	268 (72.4)	240 (80.0)	28 (40.0)	45.478[a]	＜0.001*
Yes	102 (27.6)	60 (20.0)	42 (60.0)		

Table S1: Comparison of baseline demographic and clinical characteristics between patients with and without bowel resection complications (BRC). Continuous data exhibiting a non-normal distribution are summarized as medians (IQR), while categorical variables are presented as frequencies and percentages. a: Calculated via Chi-square test. b: Calculated via Mann-Whitney U test.

Variable	Total (N = 370)	No ANC (N = 228)	ANC (N = 142)	χ2 /Z	P
Sex, n (%)					
Male	268 (72.4)	162 (71.1)	106 (74.6)	0.566[a]	0.452
Female	102 (27.6)	66 (28.9)	36 (25.4)		
Age, (Years)	28.0 (22.0, 35.2)	29.0 (21.0, 37.8)	28.0 (22.8, 33.2)	-0.661[b]	0.509
Course of diagnosis, (Months)	0.0 (0.0, 9.0)	0.0 (0.0, 7.8)	0.2 (0.0, 12.0)	-1.330[b]	0.183
Symptoms to diagnosis, (Months)	6.0 (0.5, 24.0)	6.0 (1.0, 23.0)	5.5 (0.0, 24.0)	-0.525[b]	0.599
Duration, (Months)	12.0 (4.0, 45.0)	12.0 (4.0, 36.0)	14.5 (6.0, 58.5)	-1.654[b]	0.098
Age of onset, (Years)	26.0 (20.0, 33.0)	27.0 (20.0, 35.8)	25.0 (20.0, 31.0)	-1.449[b]	0.147
BMI, (kg/m^2)	19.0 (17.3, 21.5)	19.1 (17.3, 21.5)	18.8 (17.1, 21.4)	-0.809[b]	0.419
Smoking History, n (%)					
No	323 (87.3)	203 (89.0)	120 (84.5)	1.618[a]	0.203
Yes	47 (12.7)	25 (11.0)	22 (15.5)		
Alcohol Consumption History, n (%)					
No	340 (91.9)	209 (91.7)	131 (92.3)	0.040[a]	0.841
Yes	30 (8.1)	19 (8.3)	11 (7.7)		
Hepatitis B, n (%)					
No	333 (90.0)	205 (89.9)	128 (90.1)	0.005[a]	0.943
Yes	37 (10.0)	23 (10.1)	14 (9.9)		
Abdominal History, n (%)					
No	192 (51.9)	127 (55.7)	65 (45.8)	3.454[a]	0.063
Yes	178 (48.1)	101 (44.3)	77 (54.2)		
Disease Behavior, n (%)					
No-stricturing & No-penetrating	113 (30.5)	64 (28.1)	49 (34.5)	17.656[a]	＜0.001*
Stricturing	173 (46.8)	125 (54.8)	48 (33.8)		
Penetrating	84 (22.7)	39 (17.1)	45 (31.7)		
Lesion Location, n (%)					
L1	55 (14.9)	42 (18.4)	13 (9.2)	15.675[a]	0.003*
L2	47 (12.7)	23 (10.1)	24 (16.9)		
L3	137 (37)	82 (36)	55 (38.7)		
L4	7 (1.9)	7 (3.1)	0 (0.0)		
Merge	124 (33.5)	74 (32.5)	50 (35.2)		
Diarrhea, n (%)					
No	103 (27.8)	57 (25.0)	46 (32.4)	2.382[a]	0.123
Yes	267 (72.2)	171 (75.0)	96 (67.6)		
Abdominal Pain, n (%)					
No	57 (15.4)	33 (14.5)	24 (16.9)	0.396[a]	0.529
Yes	313 (84.6)	195 (85.5)	118 (83.1)		
Abdominal Bloating, n (%)					
No	282 (76.2)	166 (72.8)	116 (81.7)	3.809[a]	0.051
Yes	88 (23.8)	62 (27.2)	26 (18.3)		
Gastrointestinal Bleeding, n (%)					
No	255 (68.9)	165 (72.4)	90 (63.4)	3.300[a]	0.069
Yes	115 (31.1)	63 (27.6)	52 (36.6)		
Weight Loss, n (%)					
No	218 (58.9)	135 (59.2)	83 (58.5)	0.021[a]	0.885
Yes	152 (41.1)	93 (40.8)	59 (41.5)		
Vomiting, n (%)					

Variable	Total (N = 370)	No ANC (N = 228)	ANC (N = 142)	χ2 /Z	P
No	321 (86.8)	199 (87.3)	122 (85.9)	0.142[a]	0.706
Yes	49 (13.2)	29 (12.7)	20 (14.1)		
Nausea, n (%)					
No	312 (84.3)	194 (85.1)	118 (83.1)	0.262[a]	0.609
Yes	58 (15.7)	34 (14.9)	24 (16.9)		
Fever, n (%)					
No	302 (81.6)	190 (83.3)	112 (78.9)	1.160[a]	0.281
Yes	68 (18.4)	38 (16.7)	30 (21.1)		
Anemia, n (%)					
None	160 (43.2)	103 (45.2)	57 (40.1)	-0.552[b]	0.581
Mild	155 (41.9)	89 (39.0)	66 (46.5)		
Moderate	50 (13.5)	34 (14.9)	16 (11.3)		
Severe	5 (1.4)	2 (0.9)	3 (2.1)		
Poor Appetite, n (%)					
No	273 (73.8)	169 (74.1)	104 (73.2)	0.035[a]	0.851
Yes	93 (26.2)	59 (25.9)	38 (26.8)		
Fatigue, n (%)					
No	303 (81.9)	191 (83.8)	112 (78.9)	1.416[a]	0.234
Yes	67 (18.1)	37 (16.2)	30 (21.1)		
Another Organ Complication History, n (%)					
No	327 (88.4)	202 (88.6)	125 (88.0)	0.028[a]	0.868
Yes	43 (11.6)	26 (11.4)	17 (12.0)		
Abdominal Complication History, n (%)					
No	308 (83.2)	184 (80.7)	124 (87.3)	2.751[a]	0.097
Yes	62 (16.8)	44 (19.3)	18 (12.7)		
Anal Complication History, n (%)					
No	162 (43.8)	127 (55.7)	35 (24.6)	34.283[a]	＜0.001[*]
Yes	208 (56.2)	101 (44.3)	107 (75.4)		
Frequency of Loose Stools	10.0 (0.0, 21.0)	12.0 (0.0, 20.0)	8.5 (0.0, 22.0)	-0.020[b]	0.984
Abdominal Pain Severity (0-3)	7.0 (2.0, 14.0)	7.0 (2.0, 14.0)	10.5 (2.0, 14.2)	-1.691[b]	0.091
General Condition (0-4)	14.0 (7.0, 17.0)	14.0 (7.0, 15.8)	14.0 (10.8, 18.0)	-3.262[b]	0.001[*]
Extramural Complications, n (%)					
0	155 (41.9)	120 (52.6)	35 (24.6)	-5.636[a]	＜0.001[*]
1	130 (35.1)	72 (31.6)	58 (40.8)		
2	72 (19.5)	31 (13.6)	41 (28.9)		
3	11 (3.0)	5 (2.2)	6 (4.2)		
4	2 (0.5)	0 (0.0)	2 (1.4)		
Opioid Antidiarrheal Medications, n (%)					
No	369 (99.7)	227 (99.6)	142 (100.0)	0.624[a]	0.429
Yes	1 (0.3)	1 (0.4)	0 (0.0)		
Reduced Hematocrit	2.0 (0.0,5.9)	1.9 (0.0,5.7)	2.5 (0.0, 6.4)	-1.235[b]	0.217
(Standard - Actual) / Standard Weight	13.0 (2.9, 22.2)	12.7 (1.7, 21.8)	13.6 (4.8, 23.0)	-1.094[b]	0.274
CDAI Score	222.8 (153.3, 260.2)	197.7 (131.1, 245.9)	233.1 (179.4, 285.4)	-4.029[b]	＜0.001[*]
Activity, n (%)					
Remission	87 (23.5)	66 (28.9)	21 (14.8)	9.754[a]	0.002[*]
Active	283 (76.5)	162 (71.1)	121 (85.2)		

Variable	Total (N = 370)	No ANC (N = 228)	ANC (N = 142)	χ2 /Z	P
WBC (×10^9)	6.6 (5.2, 8.7)	6.4 (5.1, 8.6)	6.8 (5.3, 8.9)	-1.340[b]	0.180
RBC (×10^12)	4.5 (4.0, 5.0)	4.5 (4.1, 5.0)	4.4 (4.0, 5.0)	-1.290[b]	0.197
HB (g/L)	117.5 (102.0, 133.0)	118.5 (102.0, 134.0)	117.0 (103.8, 132.0)	-0.678[b]	0.498
MCV (fL)	83.9 (78.0, 88.0)	83.7 (77.0, 87.3)	84.3 (79.0, 89.1)	-1.549[b]	0.121
MCH (Pg)	26.9 (24.2, 28.5)	26.6 (23.7, 28.4)	27.3 (24.4, 28.7)	-1.371[b]	0.170
MCHC (g/L)	318.4 (307.0, 329.0)	317.7 (305.0, 329.8)	320.0 (309.0, 328.0)	-0.829[b]	0.407
HCT (%)	36.9 (32.8, 40.9)	37.2 (32.6, 41.0)	36.4 (33.3, 40.7)	-0.769[b]	0.442
PLT (×10^9)	325.5 (256.8, 404.0)	333.0 (252.0, 402.8)	318.0 (263.2, 404.5)	-0.105[b]	0.916
ESR (mm/h)	26.6 (15.8, 47.7)	27.2 (13.9, 47.0)	26.3 (17.3, 49.3)	-1.010[b]	0.312
CRP (mg/L)	17.1 (5.0, 51.1)	17.0 (5.4, 51.8)	19.0 (4.2, 48.7)	-0.139[b]	0.890
TP (g/L)	69.7 (63.2, 75.1)	69.0 (62.7, 75.2)	70.5 (64.6, 74.8)	-1.032[b]	0.302
ALB (g/L)	37.0 (32.9, 40.6)	37.0 (32.3, 41.3)	36.9 (33.3, 40.1)	-0.561[b]	0.575
Urea Nitrogen (mmol/L)	3.7 (3.0, 4.6)	3.7 (2.9, 4.6)	3.7 (3.1, 4.6)	-0.300[b]	0.764
Serum Potassium (mmol/L)	3.9 (3.6, 4.2)	3.9 (3.7, 4.2)	3.9 (3.6, 4.2)	-0.132[b]	0.895
Uric Acid (μmol/L)	333.8 (266.5, 393.5)	342.0 (268.5, 396)	323.0 (261.4, 384.6)	-1.203[b]	0.229
HDL (mmol/L)	1.0 (0.8, 1.1)	1.0 (0.8, 1.2)	1.0 (0.8, 1.1)	-1.269[b]	0.204
Cholesterol (mmol/L)	3.5 (3.0, 4.1)	3.6 (3, 4.2)	3.4 (3.0, 4.0)	-1.667[b]	0.095
Triglycerides (mmol/L)	0.9 (0.7, 1.2)	0.9 (0.7, 1.2)	0.9 (0.7, 1.2)	-0.62[b]	0.535
Folic Acid((nmol/L))	11.5 (7.6, 16.2)	12.0 (8.7, 16.5)	10.8 (6.5, 15.3)	-2.289[b]	0.022*
VitB12 (pmmol/L)	310.7 (260.7, 384.8)	305.3 (258.7, 380.6)	324.5 (265.2, 402.5)	-1.005[b]	0.315
Fe (umol/L)	8.4 (5.2, 11.8)	9.0 (5.5, 12.2)	7.5 (4.5, 11.0)	-2.178[b]	0.029*
Total Iron-Binding Capacity (umol/L)	48.9 (42.0, 54.4)	49.5 (42.9, 55.2)	47.6 (41.0, 53.1)	-2.320[b]	0.020*
Serum Ferritin (ng/mL)	128 (66.5, 205.1)	126.4 (65.4, 200.5)	136.3 (66.5, 216.9)	-0.791[b]	0.429
Serum Transferrin (g/L)	2.0 (1.8, 2.2)	2.0 (1.8, 2.2)	2.0 (1.8 ,2.2)	-1.086[b]	0.278
Soluble Transferrin Receptor (mg/L)	1.3 (1.1, 1.6)	1.3 (1.1, 1.7)	1.3 (1.1, 1.5)	-1.383[b]	0.167
Fecal Occult Blood, n (%)					
No	74 (20.0)	49 (21.5)	25 (17.6)	0.826[a]	0.364
Yes	296 (80.0)	179 (78.5)	117 (82.4)		
Calprotectin, n (%)					
No	11 (3.0)	8 (3.5)	3 (2.1)	0.206[a]	0.650
Yes	359 (97.0)	220 (96.5)	139 (97.9)		
Fibrinogen (mg/L)	4.0 (3.2, 4.7)	4.0 (3.2, 4.7)	3.9 (3.2, 4.8)	-0.007[b]	0.994
D-Dimer (mg/L)	0.3 (0.2, 0.6)	0.3 (0.2, 0.5)	0.3 (0.2, 0.7)	-1.066[b]	0.287
Total Enteral Nutrition, n (%)					
No	268 (72.4)	178 (78.1)	90 (63.4)	9.456[a]	0.002*
Yes	102 (27.6)	50 (21.9)	52 (36.6)		

Table S2: Comparison of baseline demographic and clinical characteristics between patients with and without perianal complications (ANC). Continuous data exhibiting a non-normal distribution are summarized as medians (IQR), while categorical variables are presented as frequencies and percentages. a: Calculated via Chi-square test. b: Calculated via Mann-Whitney U test.

Variable	Total (N = 370)	No ABC (N = 286)	ABC (N = 84)	χ2 /Z	P
Sex, n (%)					
Male	268 (72.4)	210 (73.4)	58 (69.0)	0.624[a]	0.430
Female	102 (27.6)	76 (26.6)	26 (31.0)		
Age, (Years)	28.0 (22.0, 35.2)	28.0 (21.0, 35.0)	30.0 (25.0, 37.0)	-2.249[b]	0.025*
Course of diagnosis, (Months)	0.0 (0.0, 9.0)	0.0 (0.0, 6.0)	0.5 (0.0, 24.0)	-1.532[b]	0.126
Symptoms to diagnosis, (Months)	6.0 (0.5, 24.0)	6.0 (0.5, 21.0)	6.0 (1.0, 35.0)	-0.843[b]	0.399
Duration, (Years)	12.0 (4.0, 45.0)	12.0 (4.0, 36.0)	24.0 (6.1, 69.0)	-2.459[b]	0.014*
Age of onset, (Years)	26.0 (20.0, 33.0)	26.0 (20.0, 33.0)	27.5 (22.0, 36.0)	-1.697[b]	0.090
BMI, (kg/m^2)	19.0 (17.3, 21.5)	19.0 (17.3, 21.4)	19.0 (16.8, 21.6)	-0.055[b]	0.957
Smoking History, n (%)					
No	323 (87.3)	251 (87.8)	72 (85.7)	0.246[a]	0.620
Yes	47 (12.7)	35 (12.2)	12 (14.3)		
Alcohol Consumption History, n (%)					
No	340 (91.9)	262 (91.6)	78 (92.9)	0.136[a]	0.712
Yes	30 (8.1)	24 (8.4)	6 (7.1)		
Hepatitis B, n(%)					
No	333 (90.0)	255 (89.2)	78 (92.9)	0.986[a]	0.321
Yes	37 (10.0)	31 (10.8)	6 (7.1)		
Abdominal_History, n (%)					
No	192 (51.9)	151 (52.8)	41 (48.8)	0.414[a]	0.520
Yes	178 (48.1)	135 (47.2)	43 (51.2)		
Disease Behavior, n (%)					
No-stricturing & No-penetrating	113 (30.5)	99 (34.6)	14 (16.7)	13.636[a]	0.001*
Stricturing	173 (46.8)	132 (46.2)	41 (48.8)		
penetrating	84 (22.7)	55 (19.2)	29 (34.5)		
Lesion Location, n (%)					
L1	55 (14.9)	32 (11.2)	23 (27.4)	13.111[a]	0.011*
L2	47 (12.7)	36 (12.6)	11 (13.1)		
L3	137 (37)	113 (39.5)	24 (28.6)		
L4	7 (1.9)	5 (1.7)	2 (2.4)		
Merge	124 (33.5)	100 (35)	24 (28.6)		
Diarrhea, n (%)					
No	103 (27.8)	75 (26.2)	28 (33.3)	1.634[a]	0.201
Yes	267 (72.2)	211 (73.8)	56 (66.7)		
Abdominal Pain, n (%)					
No	57 (15.4)	52 (18.2)	5 (6.0)	7.451[a]	0.006*
Yes	313 (84.6)	234 (81.8)	79 (94.0)		
Abdominal Bloating, n (%)					
No	282 (76.2)	224 (78.3)	58 (69.0)	3.081[a]	0.079
Yes	88 (23.8)	62 (21.7)	26 (31.0)		
Gastrointestinal Bleeding, n (%)					
No	255 (68.9)	197 (68.9)	58 (69.0)	0.001[a]	0.977
Yes	115 (31.1)	31 (31.1)	31 (31.0)		
Weight Loss, n (%)					
No	218 (58.9)	170 (59.4)	48 (57.1)	0.142[a]	0.707
Yes	152 (41.1)	116 (40.6)	36 (42.9)		
Vomiting, n (%)					

Variable	Total (N = 370)	No ABC (N = 286)	ABC (N = 84)	χ2 /Z	P
No	321 (86.8)	259 (90.6)	62 (73.8)	15.855[a]	＜0.001[*]
Yes	49 (13.2)	27 (9.4)	22 (26.2)		
Nausea, n (%)					
No	312 (84.3)	252 (88.1)	60 (71.4)	13.672[a]	＜0.001[*]
Yes	58 (15.7)	34 (11.9)	24 (28.6)		
Fever, n (%)					
No	302 (81.6)	238 (83.2)	64 (76.2)	2.137[a]	0.144
Yes	68 (18.4)	48 (16.8)	20 (23.8)		
Anemia, n (%)					
None	160 (43.2)	130 (45.5)	30 (35.7)	-1.156[b]	0.248
Mild	155 (41.9)	113 (39.5)	42 (50.0)		
Moderate	50 (13.5)	38 (13.3)	12 (14.3)		
Severe	5 (1.4)	5 (1.7)	0 (0.0)		
Poor Appetite, n (%)					
No	273 (73.8)	220 (76.9)	53 (63.1)	6.418[a]	0.011[*]
Yes	97 (26.2)	66 (23.1)	31 (36.9)		
Fatigue, n (%)					
No	303 (81.9)	242 (84.6)	61 (72.6)	6.301[a]	0.012[*]
Yes	67 (18.1)	44 (15.4)	23 (27.4)		
Another Organ Complication History, n (%)					
No	327 (88.4)	250 (87.4)	77 (91.7)	1.144[a]	0.285
Yes	43 (11.6)	36 (12.6)	7 (8.3)		
Abdominal Complication History, n (%)					
No	308 (83.2)	257 (89.9)	51 (60.7)	39.542[a]	＜0.001[*]
Yes	62 (16.8)	29 (10.1)	33 (39.3)		
Anal Complication History, n (%)					
No	162 (43.8)	127 (44.4)	35 (41.7)	0.198[a]	0.656
Yes	208 (56.2)	159 (55.6)	49 (58.3)		
Frequency of Loose Stools	10.0 (0.0, 21.0)	10.0 (0.0, 20.2)	14.0 (0.0, 22.8)	-0.608[b]	0.543
Abdominal Pain Severity (0-3)	7.0 (2.0, 14.0)	7.0 (1.0, 14.0)	12.0 (7.0, 14.0)	-3.150[b]	0.002[*]
General Condition(0-4)	14.0 (7.0, 17.0)	14.0 (7.0, 16.0)	14.0 (10.0, 19.0)	-2.410[b]	0.016[*]
Extramural Complications, n (%)					
0	155 (41.9)	121 (42.3)	34 (40.5)	4.256[a]	0.372
1	130 (35.1)	102 (35.7)	28 (33.3)		
2	72 (19.5)	56 (19.6)	16 (19.0)		
3	11 (3.0)	6 (2.1)	5 (6.0)		
4	2 (0.5)	1 (0.3)	1 (1.2)		
Opioid Antidiarrheal Medications, n (%)					
No	369 (99.7)	286 (100.0)	83 (98.8)	0.426[a]	0.227
Yes	1 (0.3)	0 (0.0)	1 (1.2)		
Reduced Hematocrit	2.0 (0.0, 5.9)	1.8 (0.0, 5.7)	2.9 (0.0, 7.0)	-1.054[b]	0.292
(Standard - Actual) / Standard	13.0 (2.9, 22.2)	13.3 (3.0, 22.2)	11.9 (2.6, 22.2)	-0.585[b]	0.559
CDAI Score	222.8 (153.3, 260.2)	213.5 (143.6, 250.8)	234.5 (181.7, 301.6)	-3.009[b]	0.003[*]
Activity, n (%)					
Remission	87 (23.5)	74 (25.9)	13 (15.5)	3.903[a]	0.048[*]
Active	283 (76.5)	212 (74.1)	71 (84.5)		

Variable	Total (N = 370)	No ABC (N = 286)	ABC (N = 84)	χ2 /Z	P
WBC (×10^9)	6.6 (5.2, 8.7)	6.6 (5.3, 8.6)	6.5 (5.0, 8.9)	-0.221[b]	0.825
RBC (×10^12)	4.5 (4.0, 5.0)	4.5 (4.1, 5.0)	4.4 (3.8, 5.0)	-1.029[b]	0.303
HB (g/L)	117.5 (102.0, 133.0)	118.0 (103.0, 132.2)	113.0 (95.0, 134.0)	-1.442[b]	0.149
MCV (fL)	83.9 (78.0, 88.0)	84.2 (77.7, 88.2)	83.2 (78.9, 87.0)	-0.674[b]	0.500
MCH (Pg)	26.9 (24.2, 28.5)	26.9 (24.1, 28.6)	26.5 (24.4, 28.4)	-0.475[b]	0.635
MCHC (g/L)	318.4 (307.0, 329.0)	318.0 (307.0, 329.0)	319.0 (308.0, 328.8)	-0.172[b]	0.864
HCT (%)	36.9 (32.8, 40.9)	37.2 (33.3, 41.1)	35.9 (31.8, 40.6)	-1.363[b]	0.173
PLT (×10^9)	325.5 (256.8, 404.0)	325.5 (251.8, 404.5)	327.5 (273.5, 399.0)	-0.779[b]	0.436
ESR (mm/h)	26.6 (15.8, 47.7)	26.3 (14.8, 47)	28.2 (19.5, 49.6)	-1.262[b]	0.207
CRP (mg/L)	17.1(5.0, 51.1)	16.2 (4.1, 47.2)	25.3 (9.8, 59.8)	-2.416b	0.016*
TP (g/L)	69.7 (63.2, 75.1)	69.7 (63.9, 75.0)	69.0 (61.5, 75.1)	-1.224[b]	0.221
ALB (g/L)	37.0 (32.9, 40.6)	37.3 (32.9, 41.1)	35.4 (32.1, 39.9)	-2.007[b]	0.045*
Urea Nitrogen (mmol/L)	3.7 (3.0, 4.6)	3.7 (3.1, 4.6)	3.4 (2.9, 4.5)	-1.163[b]	0.245
Serum Potassium (mmol/L)	3.9 (3.6, 4.2)	3.9 (3.6, 4.1)	3.8 (3.7, 4.2)	-0.128[b]	0.898
Uric Acid (μmol/L)	333.8 (266.5, 393.5)	342.0 (272.8, 396.0)	313.6 (249.8, 369.9)	-2.263[b]	0.024*
HDL (mmol/L)	1.0 (0.8, 1.1)	1.0 (0.8, 1.2)	0.9 (0.8, 1.1)	-1.205[b]	0.228
Cholesterol (mmol/L)	3.5 (3.0, 4.1)	3.5 (3.0, 4.1)	3.5 (3.0, 4.1)	-0.388[b]	0.698
Triglycerides (mmol/L)	0.9 (0.7, 1.2)	0.9 (0.7, 1.1)	0.9 (0.8, 1.3)	-1.831[b]	0.067
Folic Acid (nmol/L)	11.5 (7.6, 16.2)	11.3 (7.9, 15.8)	13.0 (7.4, 17.1)	-0.953[b]	0.341
VitB12 (pmmol/L)	310.7 (260.7, 384.8)	314.4 (267.3, 387.5)	308.0 (230.7, 380.6)	-0.998[b]	0.318
Fe (umol/L)	8.4 (5.2, 11.8)	8.8 (5.6, 12.0)	7.4 (4.4, 10.8)	-1.890[b]	0.059
Total Iron-Binding Capacity (umol/L)	48.9 (42.0, 54.4)	49.5 (43.1, 54.9)	46.1 (39.8, 53.4)	-2.343[b]	0.019*
Serum Ferritin (ng/mL)	128.0 (66.5, 205.1)	134.1 (68.7, 213.2)	120.8 (59.1, 196.2)	-1.041[b]	0.298
Serum Transferrin (g/L)	2.0 (1.8, 2.2)	2.0 (1.8, 2.3)	1.9 (1.7, 2.2)	-1.988[b]	0.047*
Soluble Transferrin Receptor (mg/L)	1.3 (1.1, 1.6)	1.3 (1.1, 1.6)	1.3 (1.1, 1.6)	-0.045[b]	0.964
Fecal Occult Blood, n (%)					
No	74 (20.0)	61 (21.3)	13 (15.5)	1.390[a]	0.238
Yes	296 (80.0)	225 (78.7)	71 (84.5)		
Calprotectin, n (%)					
No	11 (3.0)	10 (3.5)	1 (1.2)	0.531[a]	0.466
Yes	359 (97.0)	276 (96.5)	83 (98.8)		
Fibrinogen (mg/L)	4.0 (3.2, 4.7)	3.9 (3.1, 4.7)	4.0 (3.5, 5.0)	-1.460[b]	0.144
D-Dimer (mg/L)	0.3 (0.2, 0.6)	0.3 (0.2, 0.5)	0.3 (0.2, 0.7)	-0.709[b]	0.479
Total Enteral Nutrition, n (%)					
No	268 (72.4)	226 (79.0)	42 (50.0)	27.386[a]	＜0.001*
Yes	102 (27.6)	60 (21.0)	42 (50.0)		

Table S3: Comparison of baseline demographic and clinical characteristics between patients with and without abdominal complications (ABC). Continuous data exhibiting a non-normal distribution are summarized as medians (IQR), while categorical variables are presented as frequencies and percentages. a: Calculated via Chi-square test. b: Calculated via Mann-Whitney U test.

Type	Variable	Missingness
Excluded	Ustekinumab	82.50%
	Infliximab (three categories)	29.69%
	Inadequate response	24.06%
	Infliximab dose escalation	23.13%
	Infliximab dosing interval shortening	23.13%
	Adalimumab	21.25%
	Mesalazine	20.00%
	Azathioprine	21.57%
Included	Soluble Transferrin Receptor	13.75%
	Serum Ferritin	12.19%
	Calprotectin	11.88%
	Total Iron-Binding Capacity	11.25%
	Serum Transferrin	10.94%
	Fe	10.94%
	Folic Acid	10.31%
	vitB12	10.31%
	Serum Potassium	7.81%
	Fecal Occult Blood	3.75%
	D-Dimer	2.19%
	Uric Acid	1.56%
	Triglycerides	0.94%
	Cholesterol	0.94%
	HDL	0.94%
	ESR	0.63%
	Cobblestone appearance	0.31%
	Pseudopolyps	0.31%
	CRP	0.31%
	Blood transfusion	0.31%
	Total Enteral Nutrition	0.31%
	BMI	0.31%
	Other_variables	0

Table S4. Missingness and analytical inclusion status of candidate variables. Missingness is presented as the percentage of unavailable observations. Excluded variables were not used for model development, whereas missing values in included variables were imputed within each training fold. Other variables had complete data.

Table S5. Ablation analysis of SMOTETomek and class-weighting strategies in the Random Forest models.

**Task 1 - Bowel Resection**							
**Strategy**	**AUROC**	**Accuracy**	**Sensitivity/Recall**	**Specificity**	**NPV**	**Brier score**	**ECE**
**Optimized RF pipeline[a]**	**0.86 (0.82–0.90)**	**0.78 (0.73–0.82)**	**0.83 (0.75–0.91)**	**0.76 (0.71–0.82)**	**0.95 (0.93–0.97)**	**0.115**	**0.097**
No resampling;no weighting	0.85 (0.81–0.90)	0.74 (0.68–0.80)	0.80 (0.69–0.91)	0.73 (0.65–0.80)	0.94 (0.91–0.97)	0.117	0.040
No resampling; balanced	0.86 (0.81–0.91)	0.75 (0.68–0.83)	0.79 (0.69–0.88)	0.75 (0.65–0.84)	0.94 (0.91–0.96)	0.115	0.057
No resampling; balanced_subsample	0.85 (0.80–0.90)	0.76 (0.70–0.82)	0.80 (0.70–0.90)	0.75 (0.67–0.83)	0.94 (0.92–0.97)	0.116	0.047
**Task 2 - Perianal Complications**							
**Strategy**	**AUROC**	**Accuracy**	**Sensitivity/Recall**	**Specificity**	**NPV**	**Brier score**	**ECE**
**Optimized RF pipeline[a]**	**0.71 (0.64–0.78)**	**0.68 (0.61–0.74)**	**0.64 (0.54–0.75)**	**0.70 (0.65–0.75)**	**0.76 (0.70–0.83)**	**0.212**	**0.069**
No resampling;no weighting	0.70 (0.63–0.76)	0.62 (0.58–0.66)	0.58 (0.45–0.72)	0.65 (0.58–0.72)	0.73 (0.66–0.80)	0.215	0.056
No resampling; balanced	0.70 (0.63–0.77)	0.65 (0.59–0.70)	0.66 (0.55–0.77)	0.64 (0.57–0.70)	0.76 (0.69–0.83)	0.215	0.054
No resampling; balanced_subsample	0.71 (0.64–0.78)	0.65 (0.60–0.70)	0.65 (0.52–0.78)	0.65 (0.58–0.71)	0.76 (0.69–0.83)	0.214	0.034
**Task 3 - Abdominal Complications**							
**Strategy**	**AUROC**	**Accuracy**	**Sensitivity/Recall**	**Specificity**	**NPV**	**Brier score**	**ECE**
**Optimized RF pipeline[a]**	**0.73 (0.69–0.78)**	**0.68 (0.63–0.73)**	**0.71 (0.65–0.78)**	**0.66 (0.59–0.74)**	**0.89 (0.87–0.91)**	**0.160**	**0.124**
No resampling;no weighting	0.73 (0.69–0.78)	0.72 (0.68–0.77)	0.64 (0.60–0.69)	0.74 (0.69–0.80)	0.88 (0.86–0.89)	0.159	0.056
No resampling; balanced	0.74 (0.70–0.78)	0.70 (0.67–0.73)	0.71 (0.64–0.79)	0.70 (0.64–0.76)	0.90 (0.88–0.92)	0.157	0.054
No resampling; balanced_subsample	0.73 (0.69–0.78)	0.69 (0.65–0.74)	0.71 (0.65–0.78)	0.69 (0.62–0.76)	0.89 (0.87–0.91)	0.158	0.048

Outcome	Subgroup	n	Events	Event rate, % (95% CI)	AUROC (95% CI)
**Bowel resection**	Only-SI	93	32	34.4 (25.5–44.5)	0.812 (0.720–0.894)
	Only-CI	47	6	12.8 (6.0–25.2)	0.874 (0.756–0.963)
	Others	230	32	13.9 (10.0–19.0)	0.903 (0.844–0.950)
**Perianal complications**	Only-SI	93	26	28.0 (19.9–37.8)	0.743 (0.627–0.849)
	Only-CI	47	24	51.1 (37.2–64.7)	0.681 (0.522–0.828)
	Others	230	92	40.0 (33.9–46.4)	0.700 (0.626–0.772)
**Abdominal complications**	Only-SI	93	33	35.5 (26.5–45.6)	0.703 (0.580–0.815)
	Only-CI	47	11	23.4 (13.6–37.2)	0.659 (0.492–0.813)
	Others	230	40	17.4 (13.0–22.8)	0.764 (0.681–0.839)

Table S6. Montreal phenotype–stratified sample sizes, event frequencies, complication rates, and model discrimination. Event rates are presented as events/total patients and percentages with Wilson 95% confidence intervals (CIs). AUROCs are reported with bootstrap 95% CIs.
